# Epidemiological Patterns and Variability in Acute Brain Injury: A Multicenter Registry Analysis in South Korea’s Neurocritical Care Units

**DOI:** 10.1007/s12028-025-02313-1

**Published:** 2025-07-22

**Authors:** Heewon Jeong, So Hee Park, Yoon-Hee Choo, Dong-Wan Kang, Yong Soo Kim, Bosco Seong Kyu Yang, Huimahn Alex Choi, Sung-Min Cho, Eun Jin Ha, Jiwoong Oh, Han-Gil Jeong

**Affiliations:** 1 Department of Neurosurgery, Chungnam National University Hospital, Daejeon, South Korea; 2 Department of Neurosurgery, Yeungnam University Medical Center, Daegu, South Korea; 3 Department of Neurosurgery, Seoul St. Mary’s Hospital, The Catholic University of Korea, Seoul, South Korea; 4 Division of Intensive Care Medicine, Department of Neurosurgery and Neurology, Seoul National University Bundang Hospital, Seoul National University College of Medicine, Seongnam, South Korea; 5 The NABI Institute, Department of Neurosurgery, McGovern Medical School, University of Texas Health Science Center at Houston, Houston, TX USA; 6 Division of Neurosciences Critical Care, Departments of Neurology, Surgery, Anaesthesiology and Critical Care Medicine, and Neurosurgery, Johns Hopkins University School of Medicine, Baltimore, MD USA; 7 Department of Neurosurgery and Critical Care Medicine, Seoul National University Hospital, Seoul National University College of Medicine, Seoul, South Korea; 8 Department of Neurosurgery, Severance Hospital, Yonsei University College of Medicine, Seoul, South Korea

**Keywords:** Acute brain injury, Neurocritical care, Multicenter studies, Treatment outcome

## Abstract

**Background:**

Specialized neurocritical care (NCC) improves outcomes in acute brain injury (ABI), but significant variability exists in practices across and hospitals within South Korea’s developing national NCC system. This study aims to assess clinical variability among patients with ABI across six tertiary NCC units (NCCUs) in South Korea and evaluate center-specific effects on clinical outcomes.

**Methods:**

A multicenter registry of patients with ABI admitted to NCCUs between April 2023 and April 2024 was analyzed. A descriptive analysis was conducted to evaluate demographic, clinical, and treatment characteristics across centers. Variability across centers was quantified using the average standardized mean difference (SMD) for key variables. Mixed-effects and fixed-effects models compared center-specific effects on 6- and 12-month functional outcomes (utility-weighted modified Rankin scale [mRS] score), in-hospital mortality, length of NCCU stay, and tracheostomy rates.

**Results:**

Among 1,125 patients, 202 (18.2%) had aneurysmal subarachnoid hemorrhage, 478 (42.5%) had intracerebral hemorrhage, and 442 (39.3%) had traumatic brain injury. The median Glasgow Coma Scale (GCS) score was 13 (interquartile range 7–15). Notable differences (SMD > 0.2) were observed in premorbid mRS scores, initial clinical severity (e.g., GCS, pupillary response), treatment practices (e.g. intracranial pressure monitoring, vasospasm prophylaxis), and outcomes (e.g., 6-month mRS score). Hospital-specific effects did not significantly influence most outcomes; mixed-effects models showed no significant improvement in model fit for 6-month mRS scores (*P* = 0.78), in-hospital mortality (*P* = 0.99), length of NCCU stay (*P* = 0.12), and tracheostomy rates (*P* = 0.11), except for the 12-month mRS score (*P* = 0.01).

**Conclusions:**

Significant variability exists among patients with ABI across NCCUs in South Korea. Despite these differences, center-specific effects did not significantly influence key clinical outcomes closely related to NCCU care, suggesting that variability in outcomes may be more attributable to patient-level factors.

**Supplementary information:**

The online version contains supplementary material available at 10.1007/s12028-025-02313-1.

## Introduction

Neurocritical care (NCC) is an emerging subspecialty focused on managing critically ill patients with life-threatening neurological diseases, including acute brain injury (ABI) [1]. A recent systematic review with meta-analysis has indicated that subspecialized NCC delivery is associated with reduced mortality and improved functional outcomes in patients with ABI compared to general intensive care [2]. Despite these established benefits, the adoption of specialized NCC services varies widely across health care systems, resulting in considerable variability in the scope and quality of NCC practices globally [3, 4]. Clinical practice variability is a challenge across all critical care fields, but patients with ABI add layers of complexity in the management, prognostication, and prediction of outcomes, often requiring highly individualized care approaches [5]. This complexity, compounded by challenges in applying high-evidence protocols in NCC, requires practitioners to rely on nuanced, experience-driven decision-making [6]. Although previous studies have focused largely on comparing outcomes before and after the adoption of NCC services [7], few have closely examined the variability in practices across neurocritical care units (NCCUs)—a gap that highlights the need for multicenter perspectives on ABI management.

This study aims to address this gap by analyzing a clinical registry of patients with ABI across six academic NCCUs in South Korea [8]. We sought to determine the characteristics of patients with ABI admitted to these units and assess the variability in practices across each NCCU. Additionally, we aimed to identify whether center-level characteristics are associated with patient outcomes.

## Methods

This study used prospectively collected clinical registry data from NCCUs across six tertiary academic hospitals. All participating centers operated dedicated NCCUs with 24/7 emergency services and neurosurgical capabilities. Of the six centers, four followed a semiclosed intensive care unit model, and two operated under a closed model. Each NCCU was staffed by one or two full-time neurointensivists and contained between 12 and 30 beds. Two hospitals were designated regional cardio-cerebrovascular centers (comparable to comprehensive stroke centers in the United States), and three were regional emergency medical centers. None were designated as regional trauma centers, a designation equivalent to level I trauma centers under the Korean system. Additional institutional characteristics are summarized in Supplementary Fig. 1. Six- and twelve-month outcomes were prospectively collected as part of routine quality improvement initiatives. The study period spanned from April 2023 to April 2024.

### Patient Population and Admission Criteria

The registry comprised patients admitted with ABI, specifically aneurysmal subarachnoid hemorrhage (aSAH), intracerebral hemorrhage (ICH), or traumatic brain injury (TBI). Inclusion was limited to patients presenting within 72 h of symptom onset to evaluate the acute phase management of ABI in NCCU. Admissions into NCCUs originated from three primary routes: direct visits to the emergency department (ED), transfers from other medical facilities (mostly via the ED), and in-hospital transfers from general wards.

### Data Collection

#### Demographic and Baseline Characteristics

Data on patient demographics included age, sex, and comorbidities. Upon arrival at the ED, vital signs and initial neurological status, assessed using the Glasgow Coma Scale (GCS) and pupil light reflex, were recorded. For in-hospital cases, these data were based on the time of initial discovery. Neuroimaging findings performed in the ED were also documented.

#### NCC Management and Outcomes

The retrospective data collection included details on the treatments administered from the time of hospital arrival until NCCU discharge, specifying the duration and types of therapeutic interventions. Neuromonitoring in the NCCU included transcranial Doppler in all six hospitals and automated pupillometry in five, and cerebral autoregulation monitoring was available in two hospitals. Neurological deterioration was defined as a decrease in the GCS score of 2 or more or development of pupillary abnormalities during the NCCU stay. The length of stay in the NCCU and neurological status at the time of NCCU discharge using GCS scores was noted. Additionally, the timing of hospital discharge or interdepartmental transfers was noted. Functional outcomes were evaluated at 6 and 12 months after symptom onset using the modified Rankin scale (mRS) and the Glasgow Outcome Scale Extended (GOSE).

#### Subgroup-Specific Data

Patients were categorized into three groups: aSAH, ICH, and TBI, with specific variables collected for each group. For patients with aSAH, initial assessments included the World Federation of Neurosurgical Societies (WFNS) grade, modified Fisher scale, and Hunt and Hess grading. Data on the presence and characteristics of ruptured aneurysm, hydrocephalus at presentation, and rebleeding after admission were collected. Details of both endovascular and surgical treatments were recorded, along with occurrences of neurological deterioration, vasospasm, and delayed cerebral ischemia and their corresponding treatments. For patients with ICH, data included the hemorrhage etiology, hematoma volume, the presence of acute hydrocephalus, and the modified Graeb score to quantify intraventricular hemorrhage (IVH) [9]. Information on surgical interventions, hematoma expansion, and instances of neurological deterioration during the NCCU stay was collected. For patients with TBI, data on the cause and severity of injury and findings from cranial imaging studies, such as hemorrhage location and Rotterdam computed tomography (CT) score, were provided. Details of surgical interventions were collected, along with occurrences of neurological deterioration during the NCCU stay. Additional definitions of variables used in this study are provided in the Supplemental Methods.

### Statistical Analysis

Categorical variables are described as absolute (*n*) and relative (percentage) frequencies. For numerical variables, data were expressed as either mean ± SD or median (interquartile range [IQR]) depending on their distribution. Pairwise standardized mean differences (SMDs) were calculated for variables across centers to quantify interhospital variability. The mean SMD was subsequently derived, with thresholds of > 0.5 and > 0.2 indicating moderate-to-large and small differences, respectively [10]. Statistical significance was defined as a *P* value < 0.05. We used linear or logistic mixed-effects models with a random intercept for hospitals to examine whether outcome differences (utility-weighted mRS score at 6 and 12 months; in-hospital mortality; length of NCCU stay categorized as < 3, 3–7, 7–14, 14–28, and > 28 days; and tracheostomy) were due to hospital-specific effects or patient baseline characteristics. Baseline clinical variables before NCCU admission with a bivariate *P* value < 0.05 were included as fixed effects, with hospital as a random effect. Likelihood ratio tests were used to assess the significance of the hospital-level random effect. Data analysis was performed using R statistical software, version 4.3.1 (2023–06-16). Missing data were not imputed in the analysis, ensuring the integrity of the data set as collected.

### Ethical Considerations

This study was approved by the institutional review board at each participating hospital. All procedures adhered to the ethical standards set forth by the institutional committees on human experimentation and complied with the principles outlined in the Declaration of Helsinki. The reporting of study results followed the Strengthening the Reporting of Observational Studies in Epidemiology guidelines.

## Results

### Overall Cohort

This multicenter study included 1,125 patients with ABI (42.5% ICH, 18.2% aSAH, and 39.3% TBI). The mean age was 65.8 ± 16.6 years, 45.5% were female, and the median GCS score at arrival was 13 (IQR 7–15). The median time from symptom onset to hospital arrival was 1.9 h (IQR 0.8–6.5 h). During hospitalization, 50.4% of the patients underwent cranial surgery, including endovascular treatment. Mechanical deep vein thrombosis (DVT) prophylaxis was used in 94.3%, and pharmacologic measures were administered to only 0.3%. The median length of NCCU stay was 5.5 days (IQR 2.5–13.0 days), with an NCCU mortality rate of 14.9%. A total of 467 patients (42.8%) had a GOSE score ≥ 5, and 535 patients (49.0%) had an mRS score ≤ 3 at 6 months. At 12 months, 482 patients (46.1%) had a GOSE score ≥ 5 and 528 patients (50.5%) had an mRS score ≤ 3. Additional cohort characteristics and comparison among centers are presented in Table 1.

**Table 1:** Patient characteristics compared across participating centers

	Overall (*N* = 1,125)	Hospital A (*n* = 291, 25.9%)	Hospital B (*n* = 243, 21.6%)	Hospital C (*n* = 192, 17.1%)	Hospital D (*n* = 195, 17.3%)	Hospital E (*n* = 64, 5.7%)	Hospital F (*n* = 139, 12.4%)	Average SMD	*P* value
Type of acute brain injury, *n* (%)								0.283	< 0.001
ICH	478 (42.5)	160 (55.0)	92 (37.9)	64 (33.3)	80 (41.0)	27 (42.2)	55 (39.6)		
aSAH	205 (18.2)	57 (19.6)	43 (17.7)	51 (26.6)	31 (15.9)	8 (12.5)	15 (10.8)		
TBI	442 (39.3)	74 (25.4)	108 (44.4)	77 (40.1)	84 (43.1)	29 (45.3)	69 (49.6)		
Age, mean (SD), years	65.8 (16.6)	67.1 (15.8)	64.6 (18.4)	64.8 (15.3)	65.1 (17.7)	66.0 (14.5)	67.4 (15.7)	0.090	0.36
Female, *n* (%)	512 (45.5)	136 (46.7)	110 (45.3)	93 (48.4)	82 (42.1)	31 (48.4)	59 (42.4)	0.069	0.772
Hypertension, *n* (%)	604 (53.8)	165 (56.7)	128 (52.9)	96 (50.0)	109 (56.2)	34 (54.0)	72 (51.8)	0.064	0.723
Diabetes mellitus, *n* (%)	301 (26.9)	84 (28.9)	70 (28.9)	42 (21.9)	55 (28.4)	18 (29.0)	32 (23.0)	0.083	0.421
Previous stroke, *n* (%)	187 (16.7)	63 (21.7)	50 (20.7)	11 (5.7)	31 (16.0)	13 (20.6)	19 (13.7)	0.205	< 0.001
Malignancy, *n* (%)	140 (12.5)	22 (7.6)	32 (13.2)	13 (6.8)	37 (19.1)	16 (26.7)	20 (14.4)	0.256	< 0.001
Chronic kidney disease, *n* (%)	90 (8.0)	22 (7.6)	22 (9.1)	7 (3.6)	15 (7.7)	16 (25.4)	8 (5.8)	0.247	< 0.001
Previous antiplatelet use, *n* (%)	247 (22.7)	81 (27.8)	49 (20.2)	30 (19.0)	44 (22.7)	18 (28.6)	25 (18.0)	0.130	0.093
Previous anticoagulant use, *n* (%)	152 (13.9)	29 (10.0)	26 (10.7)	50 (31.1)	25 (12.9)	10 (16.1)	12 (8.6)	0.237	< 0.001
Moderate-to-heavy alcohol use, *n* (%)	178 (15.8)	52 (17.9)	28 (11.5)	61 (31.8)	19 (9.7)	11 (17.2)	6 (4.3)	0.400	< 0.001
Current smoker, *n* (%)	217 (19.3)	63 (21.6)	41 (16.9)	43 (22.4)	31 (15.9)	13 (20.3)	26 (18.7)	0.179	0.021
Premorbid mRS ≥ 2, *n* (%)	173 (15.4)	68 (23.4)	32 (13.2)	4 (2.1)	34 (17.4)	26 (40.6)	9 (6.5)	0.628	< 0.001
Mode of admission, *n* (%)								0.368	< 0.001
ED (direct visit)	784 (69.7)	207 (71.1)	183 (75.3)	105 (54.7)	130 (66.7)	43 (67.2)	115 (82.7)		
ED (transfer from other hospital)	303 (26.9)	77 (26.5)	56 (23.0)	86 (44.8)	51 (26.2)	15 (23.4)	18 (12.9)		
In-hospital transfers (from ward)	38 (3.4)	7 (2.4)	4 (1.6)	1 (0.5)	14 (7.2)	6 (9.4)	6 (4.3)		
Time from symptom onset to arrival, median (IQR), h	1.9 (0.8–6.5)	2.0 (0.9–6.0)	1.7 (0.8–8.5)	2.1 (1.2–4.1)	2.1 (0.6–8.5)	1.8 (1.0–5.1)	1.5 (0.7–5.7)	0.149	0.648
GCS score at arrival								0.380	< 0.001
13–15, *n* (%)	585 (52.5)	189 (65.2)	140 (58.3)	69 (37.3)	73 (37.4)	29 (45.3)	84 (60.4)		
9–12, *n* (%)	190 (17.1)	56 (19.3)	35 (14.6)	43 (23.2)	30 (15.4)	10 (15.6)	16 (11.5)		
3–8, *n* (%)	339 (30.4)	45 (15.5)	65 (27.1)	73 (39.5)	92 (47.2)	25 (39.1)	39 (28.1)		
Median (IQR)	13 (7–15)	14 (11–15)	13 (8–15)	11 (6–14)	9.0 (3–14)	12 (6–14)	13 (7–15)	0.360	< 0.001
Pupillary response at arrival, *n* (%)								0.302	< 0.001
Neither one reactive	241 (21.8)	39 (13.8)	48 (20.2)	55 (29.6)	48 (25.0)	21 (32.8)	29 (20.9)		
One reactive	58 (5.3)	19 (6.7)	9 (3.8)	7 (3.8)	12 (6.2)	8 (12.5)	3 (2.2)		
Both reactive	804 (72.9)	225 (79.5)	181 (76.1)	124 (66.7)	132 (68.8)	35 (54.7)	107 (77.0)		
Systolic blood pressure at arrival, mean (SD), mm Hg	156.2 (36.1)	159.1 (34.8)	160.3 (36.4)	155.3 (38.0)	154.8 (36.0)	151.9 (38.4)	148.3 (33.4)	0.153	0.023
Diastolic blood pressure at arrival, mean (SD), mm Hg	87.5 (21.9)	88.0 (21.0)	86.0 (22.8)	94.1 (23.8)	85.9 (20.1)	85.2 (22.3)	83.6 (19.9)	0.185	< 0.001
Heart rate at arrival, mean (SD), beats/min	85.1 (21.0)	84.6 (20.1)	84.4 (21.8)	84.7 (21.3)	87.2 (20.8)	89.2 (20.2)	83.1 (21.5)	0.125	0.284
Respiratory rate at arrival, mean (SD), breaths/min	19.2 (3.7)	20.3 (3.7)	20.0 (4.1)	18.1 (2.8)	17.9 (3.2)	19.1 (4.3)	19.4 (3.7)	0.330	< 0.001
Oxygen saturation at arrival, mean (SD), %	97.0 (3.4)	97.0 (2.4)	97.6 (3.9)	96.4 (3.4)	97.0 (3.2)	97.1 (3.0)	96.7 (4.6)	0.137	0.01
Body temperature at arrival, mean (SD), °C	36.5 (1.2)	36.5 (0.7)	36.5 (0.8)	36.6 (0.9)	36.5 (1.7)	36.2 (2.8)	36.5 (0.8)	0.083	0.405
Source of NCCU admission, *n* (%)								0.271	< 0.001
ED	703 (62.5)	165 (56.7)	151 (62.1)	114 (59.4)	131 (67.2)	43 (67.2)	98 (70.5)		
Operating room	394 (35.0)	122 (41.9)	91 (37.4)	77 (40.1)	49 (25.1)	18 (28.1)	37 (26.6)		
Others	28 (2.5)	4 (1.4)	1 (0.4)	1 (0.5)	15 (7.7)	3 (4.7)	4 (2.9)		
Time from arrival to NCCU admission, median (IQR), h	4.6 (3.1–6.5)	4.3 (3.0–6.0)	5.0 (3.4–6.6)	3.4 (2.4–4.6)	5.9 (3.6–8.7)	4.4 (3.2–6.3)	5.1 (3.5–7.5)	0.214	< 0.001
Cranial surgery, *n* (%)	566 (50.4)	156 (53.6)	119 (49.0)	90 (46.9)	101 (51.8)	42 (65.6)	58 (41.7)	0.194	0.027
Symptom onset to surgery, median (IQR), h	7.6 (4.2–32.2)	6.7 (4.1–18.2)	6.8 (4.1–31.3)	4.8 (3.7–15.5)	12.0 (6.0–38.5)	11.0 (4.5–46.2)	11.1 (4.4–51.5)	0.106	0.002
Time from arrival to surgery, median (IQR), h	3.7 (2.4–11.1)	3.6 (2.6–6.4)	3.5 (2.3–5.8)	2.2 (1.7–3.5)	7.5 (3.7–19.4)	5.5 (2.9–27.8)	6.6 (3.1–23.8)	0.189	< 0.001
Intracranial pressure monitoring, *n* (%)								0.485	< 0.001
Parenchymal	23 (2.0)	10 (3.4)	3 (1.2)	0 (0.0)	0 (0.0)	10 (15.6)	0 (0.0)		
Ventricular	104 (9.2)	3 (1.0)	30 (12.3)	25 (13.0)	22 (11.3)	4 (6.2)	20 (14.4)		
Other	15 (1.3)	2 (0.7)	1 (0.4)	0 (0.0)	1 (0.5)	6 (9.4)	5 (3.6)		
Targeted temperature management, *n* (%)	119 (10.6)	15 (5.2)	28 (11.5)	19 (9.9)	27 (13.8)	21 (32.8)	9 (6.5)	0.306	< 0.001
Pharmacologic DVT prophylaxis, *n* (%)	3 (0.3)	0 (0.0)	1 (0.4)	0 (0.0)	0 (0.0)	2 (3.1)	0 (0.0)	0.106	0.001
Mechanical DVT prophylaxis, *n* (%)	1,059 (94.3)	288 (99.3)	238 (97.9)	192 (100.0)	195 (100.0)	56 (87.5)	90 (64.7)	0.488	< 0.001
Intubation, *n* (%)	547 (48.7)	114 (39.2)	134 (55.1)	89 (46.4)	112 (57.4)	47 (73.4)	51 (36.7)	0.345	< 0.001
Mechanical ventilation, *n* (%)	531 (47.2)	108 (37.1)	136 (56.0)	82 (42.7)	120 (61.5)	42 (65.6)	43 (30.9)	0.360	< 0.001
Tracheostomy, *n* (%)	139 (12.4)	26 (8.9)	31 (12.8)	12 (6.2)	40 (20.5)	16 (25.0)	14 (10.1)	0.251	< 0.001
Mortality during NCCU stay, *n* (%)	167 (14.9)	27 (9.3)	37 (15.2)	43 (22.4)	36 (18.6)	10 (15.6)	14 (10.1)	0.171	0.001
Length of NCCU stay, median (IQR), days	5.5 (2.5–13.0)	4.9 (2.8–11.9)	4.4 (1.7–11.7)	6.4 (2.6–12.3)	7.5 (3.7–16.8)	10.4 (3.6–21.2)	3.8 (1.5–9.7)	0.206	< 0.001
In-hospital mortality, *n* (%)	201 (17.9)	31 (10.8)	42 (17.3)	46 (24.0)	44 (22.7)	14 (21.9)	24 (17.3)	0.153	0.002
POLST, *n* (%)	148 (13.2)	20 (7.0)	31 (12.8)	19 (9.9)	42 (21.6)	14 (21.9)	22 (15.8)	0.217	< 0.001
mRS at 6 months 0–3, *n* (%)	535 (49.1)	154 (53.8)	130 (54.4)	77 (41.6)	88 (45.1)	15 (26.3)	71 (55.9)	0.271	< 0.001
GOSE at 6 months 5–8, *n* (%)	467 (42.9)	131 (45.8)	102 (42.7)	69 (37.3)	84 (43.1)	12 (21.1)	69 (54.3)	0.274	0.001
mRS at 12 months 0–3, *n* (%)	528 (50.5)	165 (58.3)	110 (49.8)	83 (47.7)	85 (44.5)	13 (22.8)	72 (60.5)	0.328	< 0.001
GOSE at 12 months 5–8, *n* (%)	482 (46.1)	144 (51.1)	92 (41.6)	79 (45.4)	88 (46.1)	14 (24.6)	65 (54.2)	0.248	0.002

aSAH, aneurysmal subarachnoid hemorrhage, DVT, deep vein thrombosis, ED, emergency department, GCS, Glasgow Coma Scale, GOSE, Glasgow Outcome Scale Extended, ICH, intracerebral hemorrhage, IQR, interquartile range, mRS, modified Rankin scale, NCCU, neurocritical care unit, POLST, physician orders for life-sustaining treatment, SMD, standardized mean difference, TBI, traumatic brain injury.

**Fig. 1:**
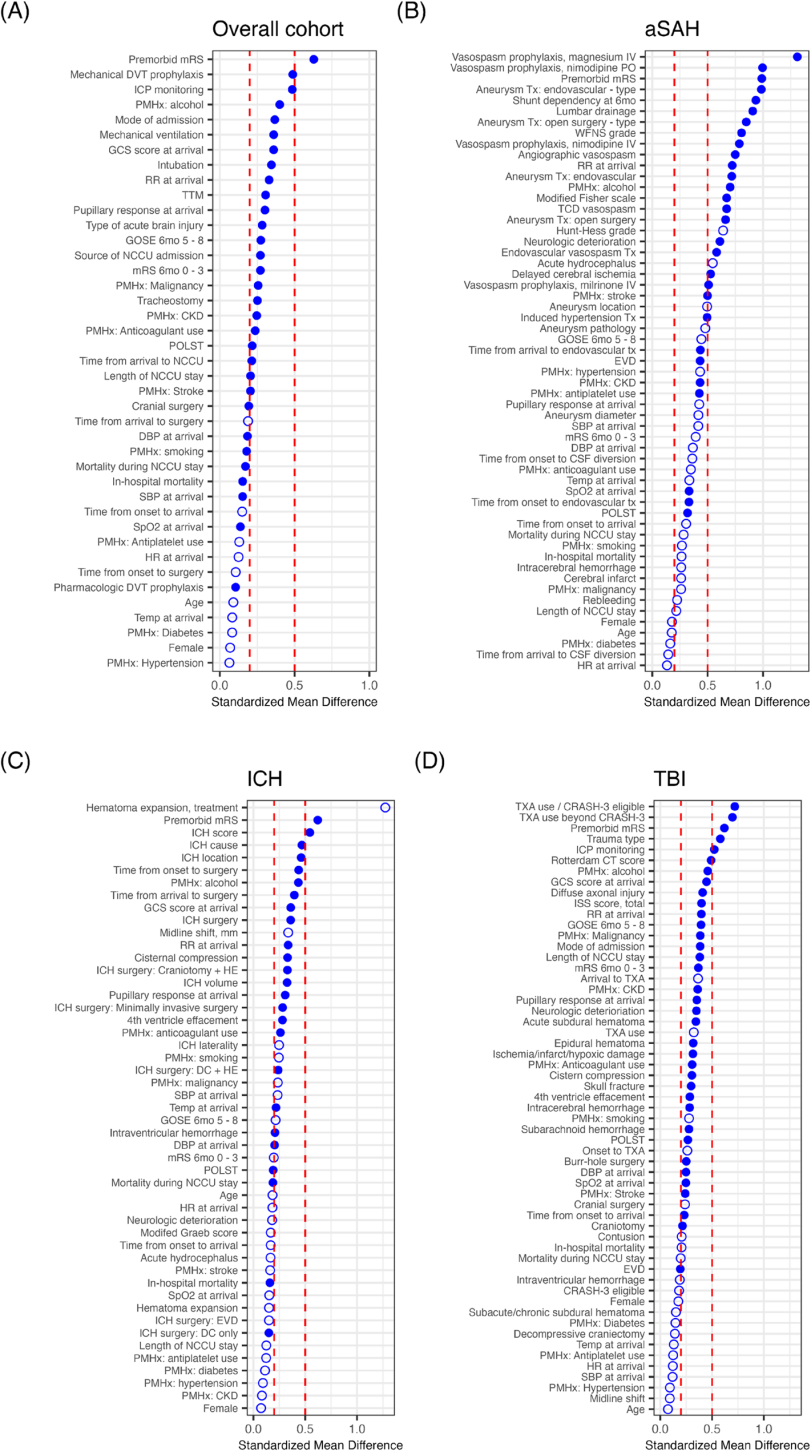
Center differences across variables measured by standardized mean difference. **a** Overall cohort. **b** aSAH. **c** ICH. **d** TBI. Filled circles: *P* < 0.05; open circles: *P* ≥ 0.05. aSAH aneurysmal subarachnoid hemorrhage, CKD chronic kidney disease, CSF cerebrospinal fluid, CT computed tomography, DBP diastolic blood pressure, DC decompressive craniectomy, DVT deep vein thrombosis, EVD external ventricular drainage, GCS Glasgow Coma Scale, GOSE Glasgow Outcome Scale Extended, HE hematoma evacuation, HR heart rate, ICH intracerebral hemorrhage, ICP intracranial pressure, ISS injury severity score, mRS modified Rankin scale, NCCU neurocritical care unit, PMHx past medical history, POLST physician orders for life-sustaining treatment, RR respiratory rate, SBP systolic blood pressure, SpO2 peripheral oxygen saturation, TBI traumatic brain injury, TCD transcranial Doppler, TTM therapeutic temperature management, Tx treatment, TXA tranexamic acid, WFNS World Federation of Neurosurgical Societies

SMD analysis revealed notable variations in baseline characteristics and interventions across centers (Fig. 1a). Premorbid mRS scores showed the largest difference, with an SMD > 0.5, indicating a substantial imbalance. Variables with moderate differences (SMD 0.2–0.5) included mechanical DVT prophylaxis, intracranial pressure (ICP) monitoring, and mode of admission. Clinical characteristics such as GCS score at arrival, pupillary response, and respiratory rate (RR) at arrival also exhibited moderate differences. Additionally, interventions such as therapeutic temperature management, mechanical ventilation, tracheostomy, and intubation demonstrated moderate variability across centers. Relevant comorbidities, including alcohol use, malignancy, chronic kidney disease, anticoagulant use, and history of stroke, also showed moderate differences. Outcome measures, such as GOSE (6 months, scores of 5–8), and advanced directives, including physician orders for life-sustaining treatment (POLST; a medical order indicating a patient’s decision to forgo specific life-sustaining treatments near the end of life), exhibited similar variability. Time from arrival to NCCU was another factor with moderate intercenter differences.

### aSAH

The mean age of patients with aSAH was 63.1 ± 14.5 years, and 69.3% were female (Supplemental Table 1). Poor-grade aSAH was observed in 40% of patients with Hunt and Hess grade IV–V and 51.2% of patients with WFNS grade IV–V. Most patients (81.9%) had a modified Fisher score of 3 or 4, and 21.7% presented with accompanying ICH. Most aneurysms (87.3%) were saccular, with common locations including the anterior communicating artery (25.0%), middle cerebral artery bifurcation (24.5%), and posterior communicating artery (20.6%). Endovascular treatment (74.1%) had a median time to treatment of 6.5 h (IQR 4.0–19.3 h), and open surgery (14.6%) was performed at a median of 12.6 h (IQR 5.7–42.9 h). NCCU mortality was 15.6%, a favorable functional outcome (mRS scores 0–3) at 6 months was achieved in 58%, and shunt dependency at 6 months was 10.4%.

Variables with the largest differences (SMD > 0.5) included premorbid mRS score, type of endovascular treatment for aneurysm, type of open surgery for aneurysm, and use of lumbar drainage (Fig. 1b). Vasospasm prophylaxis measures showed substantial differences, particularly in the use of intravenous (IV) magnesium, oral and IV nimodipine, and IV milrinone. Other variables with notable intercenter differences included shunt dependency at 6 months, angiographic vasospasm, neurologic deterioration, delayed cerebral ischemia, endovascular vasospasm therapy, and RR at arrival. Moderate differences (SMD > 0.2) were observed in the use of induced hypertension therapy, external ventricular drainage (EVD), and history of stroke or antiplatelet use. Time-related variables, such as time from onset to endovascular treatment and time from arrival to endovascular treatment, also showed moderate variability. Additional differences were noted in baseline oxygen saturation (SpO_2_) at arrival and the presence of advanced directives, such as POLST.

### ICH

The mean age of patients with ICH was 64.8 ± 16.8 years, and 47.9% were female (Supplemental Table 2). Most cases were spontaneous (84.8%), with lobar ICH (38.1%) being the most common location, followed by basal ganglia (30.5%) and thalamic ICH (17.3%). The median GCS score at arrival was 12 (IQR 7–15), and 26.4% of patients had at least one nonreactive pupil. IVH was present in 48.7% of cases, and hydrocephalus on arrival was noted in 21.3%. The median hematoma volume was 18.1 mL (IQR 6.2–43.9 mL), with 62.9% of patients classified as having small hematomas (< 30 mL). Surgical intervention was performed in 38.7%, including craniotomy (10.1%), craniectomy (9.5%), minimally invasive surgery (6.2%), and EVD (17.3%). The median time from arrival to surgery was 3.5 h (IQR 2.3–8.4 h). Mortality during NCCU admission was 15.1%, and favorable functional outcomes (mRS scores 0–3 at 6 months) were achieved in 41.2%.

Variables with the largest differences (SMD > 0.5) included premorbid mRS and ICH scores, indicating substantial variability in patient baseline conditions across centers (Fig. 1c). Moderate differences (SMD > 0.2) were observed in variables such as cause, location, and volume of ICH and pupillary response at arrival. Time-related variables, including time from onset to surgery and time from arrival to surgery, also showed moderate variability. Other clinical characteristics with notable differences included GCS score, RR, temperature, and diastolic blood pressure at arrival, as well as cisternal compression, presence of IVH, fourth ventricle effacement, and previous anticoagulant use. Surgical interventions also varied significantly, particularly in the methods used for hematoma evacuation, including craniotomy, minimally invasive surgery, and decompressive craniectomy.

### TBI

The mean age of patients with TBI was 68.1 ± 17.1 years, and 31.9% were female (Supplemental Table 3). The median time from onset to arrival was 1.9 h (IQR 0.8–6.8 h), with earlier arrivals in severe TBI cases. The median GCS score at arrival was 13 (IQR 7–15), and 25.3% had at least one nonreactive pupil, both varying significantly across hospitals. Common injury mechanisms included ground-level falls (63.6%) and road traffic accidents (12.0%). The mean injury severity score was 20.2 ± 8.0. Tranexamic acid (TXA) was administered to 51.1%, with 40.5% of these not meeting CRASH-3 trial eligibility criteria [11]. Intraparenchymal ICP monitoring was performed only in 2.3% of patients. Cranial surgery was performed in 42.0%, including 13.1% undergoing decompressive craniectomy. Neurological deterioration occurred in 24.2%, primarily due to hematoma expansion or medical complications. Mortality during NCCU admission was 14.4%, and favorable outcomes were achieved in 49.3% with mRS scores 0–3 and 42.3% with GOSE scores 5–8 at 6 months.

Variables with the largest differences (SMD > 0.5) included premorbid mRS score, type of head trauma, use of ICP monitoring, and TXA administration in both CRASH-3 eligible and ineligible patients (Fig. 1d). Notably, the proportion of CRASH-3 eligible patients showed minimal variability (SMD < 0.2). Moderate differences (SMD > 0.2) were observed in the Rotterdam CT score, injury severity score, pupillary response, RR, diastolic blood pressure, and SpO_2_ at arrival. Imaging findings, including subdural hematoma, epidural hematoma, ICH, SAH ischemic-hypoxic damage, cistern compression, skull fractures, and fourth ventricle effacement, also varied moderately. Interventions with moderate differences included burr hole surgery, craniotomy, and EVD, although decompressive craniectomy use had limited variability (SMD < 0.2). Outcome measures, such as GOSE (6 months, scores 5–8) and mRS (6 months, scores 0–3), and comorbidities, including prior alcohol use, malignancy, chronic kidney disease, stroke, and anticoagulant use, also showed moderate intercenter differences.

### Hospital-Specific Effect on Clinical Outcomes

**Fig. 2:**
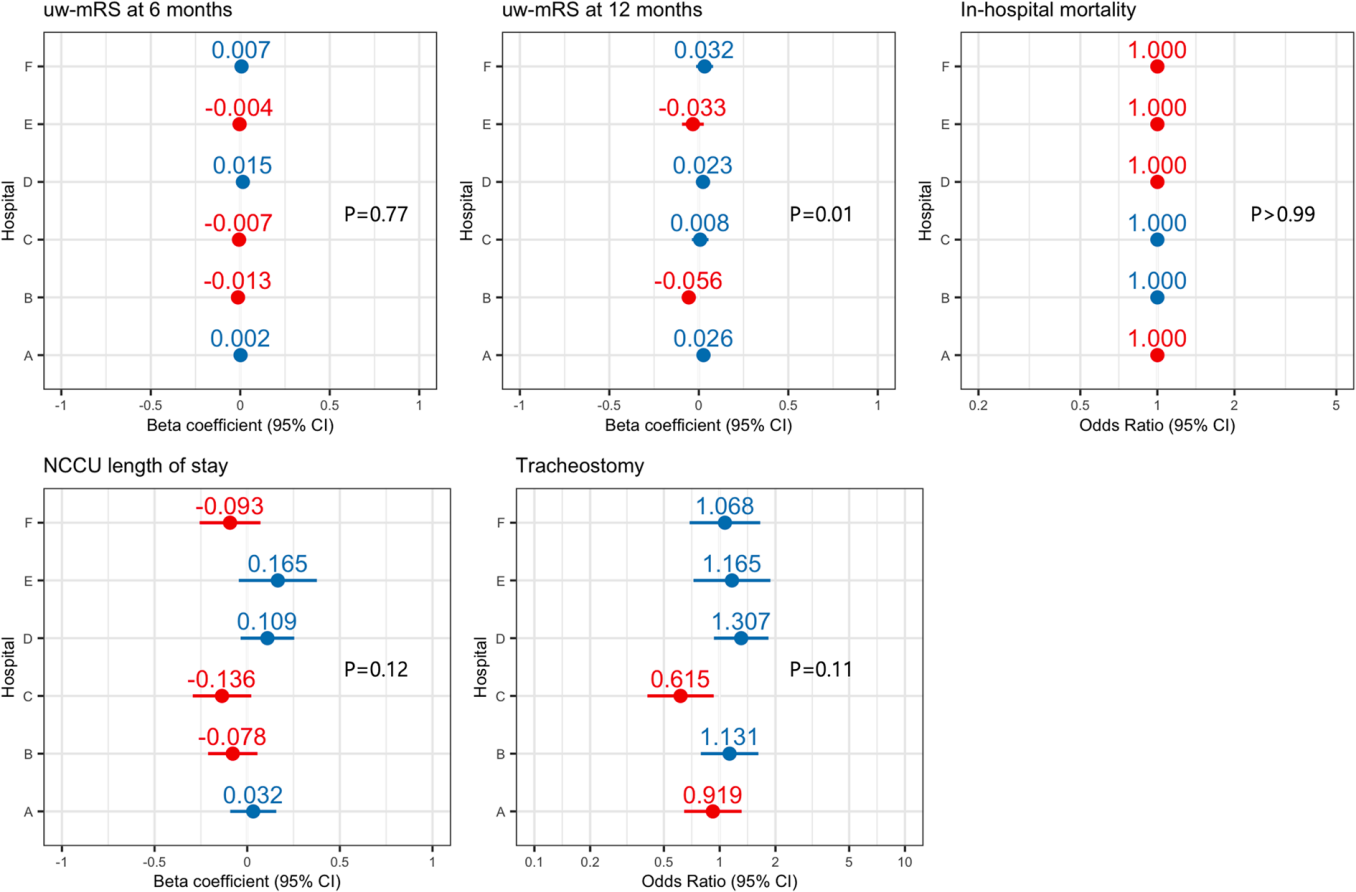
Coefficient plots for hospital-level random effects. This coefficient plot illustrates hospital-level variability across four clinical outcomes. Each point represents the effect estimate (β coefficient or odds ratio with 95% CI) for an individual hospital. The *P* value reflects the result of a likelihood ratio test comparing the mixed-effects model to the corresponding fixed-effects model. Blue dots: β coefficients > 0 or odds ratio > 1; red dots: beta coefficient ≤ 0 or odds ratio ≤ 1. CI, confidence interval; NCCU, Neurocritical Care Unit; uw-mRS, utility-weighted modified Rankin scale

We assessed the influence of hospital-specific factors on the 6-month utility-weighted mRS score, the 12-month utility-weighted mRS score, in-hospital mortality, length of NCCU stay, and tracheostomy by comparing fixed-effects and mixed-effects models with a random intercept for hospital. Adding hospital as a random effect did not significantly improve model fit for most outcomes, including the 6-month utility-weighted mRS score (*P* = 0.78), in-hospital mortality (*P* = 0.99), length of NCCU stay (*P* = 0.12), and tracheostomy (*P* = 0.11). However, for the 12-month utility-weighted mRS score, the inclusion of hospital as a random effect significantly improved model fit (*P* = 0.01), indicating a small (intraclass correlation coefficient = 0.02) but statistically significant hospital-level effect (Table 2 and Fig. 2). In contrast, baseline characteristics such as initial GCS score, pupillary response, and other clinical factors were significantly associated with outcomes. Detailed results are in Supplemental Tables 4–8.

**Table 2:** Mixed-effects and fixed-effects models for center effects on clinical outcomes

Outcome	Model	Key covariates (β, *P* value)	Random effects (τ^2^, ICC)	AIC	BIC	LRT (*P* value)
uw-mRS at 6 months	Fixed effect	Age: − 0.005 (*P* < 0.01); GCS at arrival: 0.033 (*P* < 0.01); index injury aSAH: 0.099 (*P* < 0.01); index injury TBI: 0.053 (*P* = 0.02)	None	417.8	565.0	–
	Mixed effect	Age: − 0.005 (*P* < 0.01); GCS at arrival: 0.034 (*P* < 0.01); index injury aSAH: 0.101 (*P* < 0.01); index injury TBI: 0.054 (*P* = 0.02)	τ^2^ < 0.001, ICC = 0.004	419.7	571.8	0.77
uw-mRS at 12 months	Fixed effect	Age: − 0.006 (*P* < 0.01); GCS at arrival: 0.036 (*P* < 0.01); index injury aSAH: 0.104 (*P* < 0.01); index injury TBI: 0.052 (*P* = 0.02)	None	514.9	660.7	–
	Mixed effect	Age: − 0.006 (*P* < 0.01); GCS at arrival: 0.036 (*P* < 0.01); index injury aSAH: 0.110 (*P* < 0.01); index injury TBI: 0.058 (*P* = 0.01)	τ^2^ = 0.002, ICC = 0.02	509.9	660.6	0.01
In-hospital mortality	Fixed effect	GCS at arrival: − 0.216 (*P* < 0.01); pupil reactivity at arrival, neither one reactive: 1.440 (*P* < 0.01); history of malignancy: 0.969 (*P* < 0.01)	None	668.6	772.6	–
	Mixed effect	GCS at arrival: − 0.216 (*P* < 0.01); pupil reactivity at arrival, neither one reactive: 1.440 (*P* < 0.01); history of malignancy: 0.969 (*P* < 0.01)	τ^2^ < 0.001, ICC = 0.004	670.6	779.6	> 0.99
Length of NCCU stay	Fixed effect	GCS at arrival: − 0.109 (*P* < 0.01); index injury aSAH: 0.35 (*P* < 0.01); index injury TBI: − 0.176 (*P* = 0.03)	None	3,332.7	3,431.7	–
	Mixed effect	GCS at arrival: − 0.108 (*P* < 0.01); index injury aSAH: 0.366 (*P* < 0.01); index injury TBI: − 0.156 (*P* = 0.06)	τ^2^ = 0.022, ICC = 0.015	3,332.3	3,436.3	0.12
Tracheostomy	Fixed effect	Age: − 0.015 (*P* = 0.03); GCS at arrival: − 0.196 (*p* < 0.01)	None	687.9	797.5	–
	Mixed effect	Age: − 0.015 (*P* = 0.03); GCS at arrival: − 0.190 (*P* < 0.01)	τ^2^ = 0.102, ICC = 0.03	687.4	802.0	0.11

AIC, Akaike information criterion, aSAH, aneurysmal subarachnoid hemorrhage, BIC, Bayesian information criterion, GCS, Glasgow Coma Scale, ICC, intraclass correlation coefficient, LRT, likelihood ratio test, NCCU, neurocritical care unit, TBI, traumatic brain injury, uw-mRS, utility-weighted modified Rankin scale.

## Discussion

This article presents the first descriptive report of patients with ABIs across multiple NCCUs staffed by dedicated neurointensivists in South Korea. South Korea is recognized for its high standard of health care, with one of the highest life expectancies globally and lower mortality rates from stroke compared to many other countries. However, NCC is still evolving, with significant variability in access to dedicated neurointensivists [8, 12–14]. Because the development and characteristics of NCCUs are heavily influenced by the health care system, hospital infrastructure, and local communities in which they operate, a multicenter cohort is essential to capture the broader trends and challenges, providing a more comprehensive assessment than a single-center analysis [1, 4]. Accordingly, this study identified overall outcomes, key commonalities, and interhospital heterogeneity among hospitals with neurointensivists in South Korea, providing valuable insights for future policy-making and research including multicenter clinical trials.

### Variability in the Overall Cohort and its Impacts on Outcomes

In our cohort of patients admitted to the NCCUs with ABIs, the initial GCS scores are relatively high and widely distributed. This reflects not only the admission of patients with severe conditions but also those who are monitored for potential early neurological deterioration. Additionally, this may be partly attributed to the relatively good accessibility and low-cost neurosurgery and intensive care service in South Korea [15, 16]. The median time from symptom onset to arrival at the ED is notably short, at 2 h, with direct admissions occurring within a median of 1 h, which is consistent across the centers in our cohort, with no significant intercenter variability. This can be explained by the well-developed emergency medical services systems in South Korea, where the transport and initial treatment of suspected stroke patients are highly advanced, and the high population density allows for short travel times to hospitals [17]. A unique aspect of our cohort is the high use of mechanical DVT prophylaxis (intermittent pneumatic compression) in the NCCU, whereas the use of pharmacologic DVT prophylaxis remains very low. The incidence of DVT and pulmonary embolism is known to be lower in East Asian populations [18, 19], and these patients are more susceptible to bleeding [20]. As a result, there is a strong reluctance to use anticoagulants in the NCCU, particularly in patients with recent intracranial hemorrhage or those who have undergone neurosurgical procedures. However, the risks and benefits of this strategy in the Korean NCCU population need to be further investigated through prospective studies.

Although all participating hospitals are university affiliated, there were significant interhospital differences in variables. These differences are likely due to characteristics of the local community and each hospital’s unique role. Hospitals A and B are designated as regional cardio-cerebrovascular centers, thus admitting a higher number of regional severe stroke patients. Hospital E is a large institution that serves as a national referral center for rare diseases, resulting in a lower number of patients with ABI relative to its size but a higher proportion of patients with cancer and those with poor prehospital health conditions. In the Korean health care system, tertiary academic hospitals often serve as de facto referral centers for severe stroke and trauma, even without formal designation as trauma or stroke centers. Therefore, despite differences in case mix, interhospital comparison remains meaningful within this decentralized but functionally capable system. There was variability in the neurological severity of patients upon arrival, which contributed to the differences in characteristics of the patient populations that each hospital serves and differences in NCCU admission criteria. Consequently, in-hospital mortality (range 10.8–24.0%) and 6-month favorable functional outcomes (mRS scores 0–3, range 26.3–55.9%) showed considerable heterogeneity across hospitals. However, there is no statistically significant difference in comparison between mixed-effects and fixed-effects models, suggesting that the differences in functional outcomes and in-hospital mortality were primarily influenced by individual patient factors rather than hospital-specific characteristics. However, the 12-month outcome revealed a small but statistically significant hospital effect. This suggests that longer-term recovery may be influenced by postdischarge factors, such as access to rehabilitation or outpatient services, which warrant further investigation.

### Variability in Characteristics and Management of aSAH

This multicenter Korean NCCU study highlights unique characteristics in the management of aSAH, particularly the efficiency of treatment timelines compared to international cohorts. The expedited processes observed in Korean NCC reflect a well-coordinated system, encompassing rapid emergency response, effective prehospital triage, and specialized care. This efficiency is particularly relevant for poor-grade aSAH, in which timely intervention is critical to reducing complications and improving outcomes. The cohort demonstrated shorter treatment timelines than those typically reported in Western populations, with prompt aneurysm management likely contributing to reduced risks of complications such as delayed cerebral ischemia and vasospasm. Although endovascular treatment was the dominant modality across most centers, the variability in the use of surgical clipping highlights the influence of institutional expertise and resource availability, which may shape treatment decisions.

However, interhospital variability appeared most prominent in patients with aSAH. This pattern was less pronounced in patients with ICH or TBI, suggesting that institutional preferences and protocols may have a greater influence on the management of aSAH. The management of acute hydrocephalus in aSAH showed variability in cerebrospinal fluid diversion strategies, with EVD more commonly employed in severe cases and lumbar drainage in less severe cases. Notably, there were significant differences in the preferred strategies across centers, highlighting the influence of institutional practices on treatment approaches. Similarly, strategies for vasospasm prophylaxis, such as nimodipine administration (IV [range 10.7–86.0%] vs. oral [range 0–85.7%]), IV magnesium (range 0–86.0%), and lumbar drainage (range 2–75%), also differed substantially across centers [21]. These patterns reflect patient-specific decision-making based on the clinical condition of patients. The relatively low rates of complications, such as vasospasm and infarction, and favorable functional outcomes in many patients suggest that expedited intervention and preventive measures play an important role in patient recovery. However, it is important to acknowledge the potential limitations of this clinical registry, including the possibility of a low outcome capture rate compared with a prospective cohort or randomized controlled study, which could affect the accuracy of the reported results.

### Variability in Characteristics and Management of ICH and TBI

The volume of patients with ICH treated varied significantly across hospitals, with hospitals A and B, both designated as regional cardio-cerebrovascular centers, managing a higher proportion of these cases. The severity of patients admitted to the NCCU, reflected in ICH score, ICH volume, and GCS score at arrival, also varied across centers, which may be attributed to differences in operational policies between hospitals and local patient characteristics. For instance, some hospitals operate without a step-down unit, potentially influencing the admission of patients with relatively lower severity to the NCCU. Variations were also observed in onset-to-surgery times between centers, reflecting differences in patient referral patterns and regional health care infrastructure. For instance, hospital E demonstrated longer onset-to-arrival and onset-to-surgery times, likely due to a higher proportion of patients being transferred from other facilities and regions. Despite these differences, a shared emphasis of rapid surgical intervention was evident across all centers, with an average onset-to-surgery time of 5.8 h [IQR 3.8–14.0] [22–24]. The method of surgical interventions, such as decompressive craniectomy and minimally invasive surgery, also varied significantly. Because these data predate the recent publication of a successful randomized controlled trial on minimally invasive surgery, it will be important to assess whether treatment trends in South Korea have shifted following this development [25].

Despite not being designated as regional trauma centers, the participating hospitals in this study managed a substantial number of TBI cases, including severe neurotrauma. The cohort included a significant proportion of such cases, with notable center-specific differences in treatment approaches. The predominance of ground-level falls as an injury mechanism reflects the aging population and highlights the need for preventive strategies tailored to older adults [26]. Polytrauma cases were less frequent because even in the absence of regional trauma centers in our cohort [27]. Variability in treatment strategies, such as the use of TXA and surgical interventions, underscores institutional preferences and resource availability. The discrepancy in TXA administration, with some eligible patients untreated and others outside CRASH-3 criteria treated, highlights the need for nationwide efforts to standardize and enforce evidence-based protocols in clinical practice. Despite guideline recommendations, intraparenchymal ICP monitoring remains underused in Korea. This is likely due to structural limitations within the universal health care system, including low reimbursement for devices with limited high-level evidence, which has led to restricted availability of ICP monitoring equipment. This represents a distinctive challenge in Korea’s health care system, where certain specialized NCC practices remain underused. Although debates over its universal efficacy persist, recent studies demonstrate its continued importance and potential benefits [28, 29]. Given this evidence, restrictive use based solely on reimbursement or efficacy concerns warrants reconsideration.

### Limitations

First, the absence of regional trauma centers from participating centers resulted in a lower proportion of polytrauma patients and a generally lower severity of TBI [30]. Second, although the six tertiary hospitals are distributed across the country, the generalizability of the findings is not guaranteed given the unique characteristics of each hospital and patient population. Third, this study focused on patients with TBI, aSAH, and ICH because most centers in this consortium operate neurosurgery-based NCCUs, where these conditions account for the majority of admissions. In South Korea, NCCUs are typically department-led rather than combined, and patients ischemic stroke—especially those not requiring decompressive surgery—are usually managed by neurology-based stroke units or NCCUs. Fourth, data on the specific treatments provided in the NCCU for each patient and the causes of mortality were limited, which may influence the interpretation of mortality outcomes across hospitals. Several treatment practices in our cohort diverged from international guidelines, including limited ICP monitoring, variability in aSAH management, and low pharmacological VTE.

prophylaxis. These differences may reflect national guidelines, health care system constraints, and population-specific factors, and could have contributed to the lack of interhospital outcome variation. Differences in neuromonitoring practices across hospitals may have influenced patient outcomes, although these variations were not systematically quantified in this study. Fifth, we need to provide cognitive or behavioral outcomes of patients [31, 32]. Sixth, due to the small sample size in certain hospitals, particularly in subgroup analyses, the low number of patients in specific centers may have disproportionately influenced the average SMD. Finally, the lack of significant differences in outcomes between hospitals should not be interpreted as an indication that there are no areas for improvement in care delivery, either at the individual hospital level or across the system as a whole.

## Conclusions

This study is the first to present multicenter registry data from Korean NCCUs, providing a comprehensive analysis of patients with ABI and highlighting significant variability in practices across six academic NCCUs. Our findings demonstrate that although patient-specific factors were the primary drivers of outcomes, center-specific differences in clinical management underline the need for greater harmonization of care protocols. Establishing this collaborative research consortium has created a robust foundation for future research and clinical trials.

## Supplementary information

Below is the link to the electronic supplementary material.

Supplementary file1 (DOCX 398 kb)
