# Supplementary material for: Epidemiological Patterns and Variability in Acute Brain Injury: A Multicenter Registry Analysis in South Korea’s Neurocritical Care Units
Source: Neurocrit Care. 2025 Jul 22;43(3):810–23. doi: 10.1007/s12028-025-02313-1 (PMC12647344; doi:10.1007/s12028-025-02313-1)
Supplement: Supplementary file 1 — Supplementary file1 (DOCX 398 kb) [file 12028_2025_2313_MOESM1_ESM.docx]

**Supplemental Materials:**

**Epidemiological Patterns and Variability in Acute Brain Injury: A Multicenter Cohort Analysis in South Korea’s Neurocritical Care Units**

Heewon Jeong^1†^, So Hee Park^2†^, Yoon Hee Choo^3†^, Dong-Wan Kang^4^, Yong Soo Kim^4^, Bosco Seong Kyu Yang^5^, Huimahn Alex Choi^5^, Sung-Min Cho^6^, Eun Jin Ha^7‡^, Jiwoong Oh^8‡^, Han-Gil Jeong^4‡^ on behalf of Neurocritical Care Alliance for Research and Trials - Korea (NCART-KR)

1 Department of Neurosurgery, Chungnam National University Hospital

2 Department of Neurosurgery, Yeungnam University Medical Center

3 Department of Neurosurgery, Seoul St. Mary’s Hospital, The Catholic University of Korea

4 Division of Intensive Care Medicine, Department of Neurosurgery and Neurology, Seoul National University Bundang Hospital, Seoul National University College of Medicine

5 The NABI institute, Department of Neurosurgery, McGovern Medical School, University of Texas Health Science Center at Houston, Houston, TX, USA

6 Division of Neurosciences Critical Care, Departments of Neurology, Surgery, Anaesthesiology and Critical Care Medicine and Neurosurgery, Johns Hopkins University School of Medicine, Baltimore, USA

7 Department of Neurosurgery and Critical Care Medicine, Seoul National University Hospital, Seoul National University College of Medicine

8 Department of Neurosurgery, Severance Hospital, Yonsei University College of Medicine

† These authors contributed equally to this work and are considered co-first authors.

‡ These authors contributed equally to this work and are considered co-corresponding authors.

**Methods**

**Definitions of key variables**

**Comorbidities and Lifestyle Factors**

Chronic kidney disease (CKD) was defined as stage III or higher. Alcohol consumption was categorized as none (no alcohol consumption in the past year), infrequent drinker (1–11 drinks in the past year), light drinker (≥12 drinks in the past year but ≤3 drinks per week), moderate drinker (>3 drinks per week to ≤7 drinks per week for women and >3 drinks per week to ≤14 drinks per week for men), and heavy drinker (>7 drinks per week for women and >14 drinks per week for men).

**Intracerebral Hemorrhage**

Cranial imaging findings were assessed using the initial CT scan obtained in the emergency room, evaluating hemorrhage location, volume, cisternal compression, fourth ventricle effacement, acute hydrocephalus, and midline shift. Hematoma volume was measured using the ABC/2 method,[^1^](https://www.zotero.org/google-docs/?eCSxBX) and intraventricular hemorrhage severity was assessed using the modified Graeb Score.[^2^](https://www.zotero.org/google-docs/?jYC135) The ICH score was calculated based on Hemphill et al.[^3^](https://www.zotero.org/google-docs/?aOtNrA) Hematoma expansion was defined as an increase in volume by >6 mL or >33% on follow-up imaging.[^4^](https://www.zotero.org/google-docs/?FoNfVL) Surgical interventions were categorized as decompressive craniectomy alone, craniectomy with hematoma evacuation, craniotomy with hematoma evacuation, and minimally invasive surgery (MIS) or external ventricular drainage (EVD). MIS was defined as endoscopic or stereotactic hematoma evacuation or intrahematoma catheter insertion, with or without thrombolysis.

**Aneurysmal Subarachnoid Hemorrhage**

Rebleeding was defined as sudden clinical deterioration with increased hemorrhage on CT compared to prior imaging or confirmation at autopsy, sudden deterioration with fresh blood observed in a ventricular drain when no imaging or autopsy was available, active contrast extravasation on angiography, or intraoperative rupture before aneurysm clipping.

Aneurysms were classified morphologically as saccular, dissecting, or fusiform. Locations included cavernous, persistent trigeminal, paraclinoid (medial and lateral), ophthalmic, superior hypophyseal, posterior communicating, anterior choroidal, internal carotid bifurcation, middle cerebral bifurcation, anterior communicating, pericallosal, posterior inferior cerebellar artery, superior cerebellar, basilar apex, and others. Endovascular treatments were categorized as coiling, balloon-assisted coiling, stent alone, stent-assisted coiling, vessel occlusion, flow diverter, other, and none/attempted. Open surgical procedures included bypass, clipping, trapping alone, wrapping, Hunterian ligation alone, other, and none/attempted. Cases involving both bypass and trapping were classified as bypass. The “none/attempted” category included cases where treatment was attempted but not completed.

Delayed cerebral ischemia (DCI) was defined according to Vergouwen et al.[^5^](https://www.zotero.org/google-docs/?dgQULF) and included clinical and functional DCI. Clinical DCI was defined as new focal neurological impairment (e.g., hemiparesis, aphasia, apraxia, hemianopia, neglect) or a ≥2-point decrease in the Glasgow Coma Scale (total or individual component) persisting for ≥1 hour, occurring beyond aneurysm occlusion, and not attributable to other causes based on clinical, imaging, and laboratory assessments. Functional DCI, applicable to unconscious patients, was defined as a perfusion deficit with a typical cerebral blood flow/mean transit time mismatch occurring >72 hours after ictus, with an MTT exceeding 5.0–6.4 seconds or regional blood flow <25–40 ml/100 g/min.

**Traumatic Brain Injury**

Injury causes were categorized as ground-level fall, fall from height (>1 meter), road traffic accident, assault, unknown, and other. Severity was assessed using the Injury Severity Score (ISS)[^6^](https://www.zotero.org/google-docs/?CIXmSN) and the Abbreviated Injury Score (AIS) for different body regions, including head/neck, face, chest, abdomen, extremities, and external areas.[^7^](https://www.zotero.org/google-docs/?6d5Hcc) Cranial imaging findings were assessed using the initial CT scan and included epidural hematoma, subdural hematoma (acute or chronic), subarachnoid hemorrhage, skull fractures, contusions, intracerebral hemorrhage, intraventricular hemorrhage, cisternal compression, fourth ventricle effacement, diffuse axonal injury, and ischemic or hypoxic damage. The Rotterdam CT score was calculated,[^8^](https://www.zotero.org/google-docs/?kBkIn1) and midline shift was recorded.

**References**

[1. Kothari RU, Brott T, Broderick JP, et al. The ABCs of Measuring Intracerebral Hemorrhage Volumes. *Stroke*. 1996;27(8):1304-1305. doi:10.1161/01.STR.27.8.1304](https://www.zotero.org/google-docs/?OxeFzw)

[2. Morgan TC, Dawson J, Spengler D, et al. The Modified Graeb Score: an enhanced tool for intraventricular hemorrhage measurement and prediction of functional outcome. *Stroke*. 2013;44(3):635-641. doi:10.1161/STROKEAHA.112.670653](https://www.zotero.org/google-docs/?OxeFzw)

[3. Hemphill JC, Bonovich DC, Besmertis L, Manley GT, Johnston SC. The ICH score: a simple, reliable grading scale for intracerebral hemorrhage. *Stroke*. 2001;32(4):891-897. doi:10.1161/01.str.32.4.891](https://www.zotero.org/google-docs/?OxeFzw)

[4. Sprigg N, Flaherty K, Appleton JP, et al. Tranexamic acid for hyperacute primary IntraCerebral Haemorrhage (TICH-2): an international randomised, placebo-controlled, phase 3 superiority trial. *Lancet*. 2018;391(10135):2107-2115. doi:10.1016/S0140-6736(18)31033-X](https://www.zotero.org/google-docs/?OxeFzw)

[5. Vergouwen MDI, Vermeulen M, van Gijn J, et al. Definition of delayed cerebral ischemia after aneurysmal subarachnoid hemorrhage as an outcome event in clinical trials and observational studies: proposal of a multidisciplinary research group. *Stroke*. 2010;41(10):2391-2395. doi:10.1161/STROKEAHA.110.589275](https://www.zotero.org/google-docs/?OxeFzw)

[6. Baker SP, O’Neill B. The injury severity score: an update. *J Trauma*. 1976;16(11):882-885. doi:10.1097/00005373-197611000-00006](https://www.zotero.org/google-docs/?OxeFzw)

[7. Gennarelli TA, Wodzin E. AIS 2005: a contemporary injury scale. *Injury*. 2006;37(12):1083-1091. doi:10.1016/j.injury.2006.07.009](https://www.zotero.org/google-docs/?OxeFzw)

[8. Maas AIR, Hukkelhoven CWPM, Marshall LF, Steyerberg EW. Prediction of outcome in traumatic brain injury with computed tomographic characteristics: a comparison between the computed tomographic classification and combinations of computed tomographic predictors. *Neurosurgery*. 2005;57(6):1173-1182; discussion 1173-1182. doi:10.1227/01.neu.0000186013.63046.6b](https://www.zotero.org/google-docs/?OxeFzw)

**Supplemental Figure 1. Characteristics and Locations of Participating Centers**


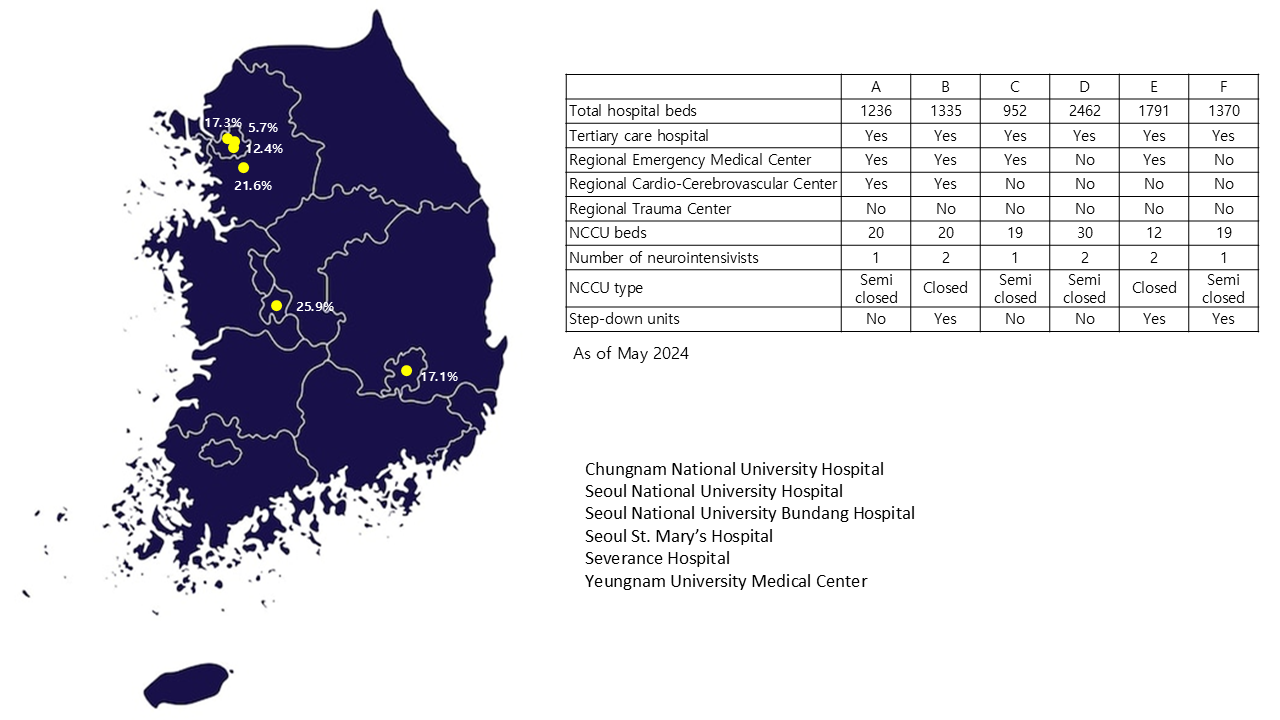


**Supplemental Table 1. aSAH Patient Characteristics Compared Across Participating Centers**

|  | Overall (n=205) | Hospital A (n=57,  27.8%) | Hospital B (n=43,  21.0%) | Hospital C (n=51, 24.9%) | Hospital D (n=31, 15.1%) | Hospital E (n=8, 3.9%) | Hospital F (n=15, 7.3%) | SMD | P-value |
| --- | --- | --- | --- | --- | --- | --- | --- | --- | --- |
| Age, years | 63.1 (14.5) | 64.6 (12.8) | 62.7 (17.0) | 61.4 (14.6) | 65.0 (14.4) | 63.2 (14.9) | 59.5 (13.6) | 0.176 | 0.729 |
| Female | 142 (69.3) | 36 ( 63.2) | 29 ( 67.4) | 37 ( 72.5) | 25 ( 80.6) | 5 ( 62.5) | 10 ( 66.7) | 0.178 | 0.634 |
| Hypertension | 104 (50.7) | 30 ( 52.6) | 25 ( 58.1) | 19 ( 37.3) | 16 ( 51.6) | 7 ( 87.5) | 7 ( 46.7) | 0.432 | 0.103 |
| Diabete mellitus | 36 (17.6) | 11 ( 19.3) | 11 ( 25.6) | 6 ( 11.8) | 5 ( 16.1) | 1 ( 12.5) | 2 ( 13.3) | 0.163 | 0.611 |
| Previous stroke | 19 ( 9.3) | 7 ( 12.3) | 7 ( 16.3) | 1 ( 2.0) | 0 ( 0.0) | 3 ( 37.5) | 1 ( 6.7) | 0.500 | 0.004 |
| Malignancy | 15 ( 7.3) | 3 ( 5.3) | 1 ( 2.3) | 5 ( 9.8) | 2 ( 6.5) | 1 ( 12.5) | 3 ( 20.0) | 0.260 | 0.277 |
| Chronic kidney disease | 10 ( 4.9) | 3 ( 5.3) | 2 ( 4.7) | 1 ( 2.0) | 1 ( 3.2) | 3 ( 37.5) | 0 ( 0.0) | 0.432 | 0.001 |
| Previous antiplatelet use | 27 (13.5) | 11 ( 19.3) | 4 ( 9.3) | 4 ( 8.7) | 3 ( 9.7) | 4 ( 50.0) | 1 ( 6.7) | 0.425 | 0.021 |
| Previous anticoagulant use | 13 ( 6.5) | 4 ( 7.0) | 1 ( 2.3) | 7 ( 15.2) | 0 ( 0.0) | 1 ( 14.3) | 0 ( 0.0) | 0.349 | 0.054 |
| Moderate-to-heavy alcohol use | 25 (12.2) | 6 ( 10.5) | 5 ( 11.6) | 13 ( 25.5) | 0 ( 0.0) | 1 ( 12.5) | 0 ( 0.0) | 0.703 | 0.001 |
| Current smoker | 38 (18.5) | 12 ( 21.1) | 8 ( 18.6) | 10 ( 19.6) | 3 ( 9.7) | 2 ( 25.0) | 3 ( 20.0) | 0.268 | 0.911 |
| Premorbid mRS ≥2 | 16 ( 7.8) | 6 ( 10.5) | 2 ( 4.7) | 0 ( 0.0) | 3 ( 9.7) | 5 ( 62.5) | 0 ( 0.0) | 0.989 | <0.001 |
| Mode of admission |  |  |  |  |  |  |  | 0.636 | 0.002 |
| Direct to ER | 106 (51.7) | 28 ( 49.1) | 29 ( 67.4) | 16 ( 31.4) | 15 ( 48.4) | 5 ( 62.5) | 13 ( 86.7) |  |  |
| From other hospital to ER | 6 ( 2.9) | 3 ( 5.3) | 1 ( 2.3) | 0 ( 0.0) | 1 ( 3.2) | 1 ( 12.5) | 0 ( 0.0) |  |  |
| In-hospital (except ER) | 93 (45.4) | 26 ( 45.6) | 13 ( 30.2) | 35 ( 68.6) | 15 ( 48.4) | 2 ( 25.0) | 2 ( 13.3) |  |  |
| Time from symptom onset to arrival, h | 2.3 [0.9, 7.6] | 3.3 [1.0, 8.4] | 1.2 [0.7, 8.4] | 2.4 [2.0, 5.2] | 3.6 [0.7, 8.4] | 1.4 [0.9, 4.4] | 1.1 [0.5, 2.0] | 0.306 | 0.149 |
| GCS score at arrival |  |  |  |  |  |  |  | 0.549 | 0.010 |
| 13 - 15 | 105 (52.0) | 38 ( 66.7) | 25 ( 58.1) | 18 ( 37.5) | 13 ( 41.9) | 3 ( 37.5) | 8 ( 53.3) |  |  |
| 9 - 12 | 27 (13.4) | 7 ( 12.3) | 4 ( 9.3) | 11 ( 22.9) | 1 ( 3.2) | 3 ( 37.5) | 1 ( 6.7) |  |  |
| 3 - 8 | 70 (34.7) | 12 ( 21.1) | 14 ( 32.6) | 19 ( 39.6) | 17 ( 54.8) | 2 ( 25.0) | 6 ( 40.0) |  |  |
| Median [IQR] | 13.0 [6.0, 15.0] | 15.0 [12.0, 15.0] | 13.0 [7.0, 15.0] | 11.0 [4.8, 14.0] | 6.0 [3.0, 14.0] | 12.0 [7.5, 13.5] | 13.0 [4.5, 14.0] | 0.340 | 0.002 |
| Pupillary response at arrival |  |  |  |  |  |  |  | 0.424 | 0.085 |
| neither one reactive | 58 (28.7) | 9 ( 16.1) | 10 ( 23.3) | 21 ( 42.0) | 10 ( 33.3) | 2 ( 25.0) | 6 ( 40.0) |  |  |
| one reactive | 8 ( 4.0) | 2 ( 3.6) | 1 ( 2.3) | 1 ( 2.0) | 3 ( 10.0) | 1 ( 12.5) | 0 ( 0.0) |  |  |
| both reactive | 136 (67.3) | 45 ( 80.4) | 32 ( 74.4) | 28 ( 56.0) | 17 ( 56.7) | 5 ( 62.5) | 9 ( 60.0) |  |  |
| Systolic blood pressure at arrival, mmHg | 154.3 (36.7) | 153.4 (35.5) | 162.7 (38.7) | 146.2 (33.8) | 158.9 (42.0) | 171.5 (16.0) | 142.5 (35.2) | 0.415 | 0.126 |
| Diastolic blood pressure at arrival, mmHg | 88.6 (23.5) | 87.6 (23.8) | 85.0 (27.5) | 91.5 (21.5) | 90.6 (23.2) | 100.4 (11.7) | 82.8 (20.4) | 0.367 | 0.414 |
| Heart rate at arrival, beats/min | 84.4 (24.4) | 82.3 (19.9) | 82.1 (26.1) | 86.2 (25.2) | 88.6 (27.4) | 87.5 (30.9) | 82.8 (24.1) | 0.133 | 0.824 |
| Respiratory rate at arrival, breaths/min | 18.8 (3.2) | 20.2 (3.4) | 20.0 (2.9) | 17.7 (3.0) | 17.2 (2.5) | 16.5 (2.3) | 19.1 (1.6) | 0.722 | <0.001 |
| Oxygen saturation at arrival, % | 97.1 (4.0) | 97.1 (2.8) | 97.9 (2.8) | 96.8 (3.8) | 98.1 (1.9) | 97.5 (2.4) | 93.6 (10.3) | 0.333 | 0.011 |
| Body temperature at arrival, °C | 36.5 (0.8) | 36.5 (0.8) | 36.3 (0.9) | 36.5 (0.8) | 36.8 (0.9) | 36.4 (0.4) | 36.1 (0.8) | 0.335 | 0.145 |
| Hunt-Hess grade |  |  |  |  |  |  |  | 0.640 | 0.403 |
| 1 | 31 (15.1) | 13 ( 22.8) | 7 ( 16.3) | 6 ( 11.8) | 4 ( 12.9) | 0 ( 0.0) | 1 ( 6.7) |  |  |
| 2 | 55 (26.8) | 15 ( 26.3) | 12 ( 27.9) | 15 ( 29.4) | 8 ( 25.8) | 1 ( 12.5) | 4 ( 26.7) |  |  |
| 3 | 37 (18.0) | 11 ( 19.3) | 10 ( 23.3) | 9 ( 17.6) | 1 ( 3.2) | 3 ( 37.5) | 3 ( 20.0) |  |  |
| 4 | 37 (18.0) | 11 ( 19.3) | 5 ( 11.6) | 11 ( 21.6) | 6 ( 19.4) | 2 ( 25.0) | 2 ( 13.3) |  |  |
| 5 | 45 (22.0) | 7 ( 12.3) | 9 ( 20.9) | 10 ( 19.6) | 12 ( 38.7) | 2 ( 25.0) | 5 ( 33.3) |  |  |
| WFNS grade |  |  |  |  |  |  |  | 0.806 | 0.003 |
| Grade 1 | 55 (26.8) | 22 ( 38.6) | 16 ( 37.2) | 7 ( 13.7) | 8 ( 25.8) | 1 ( 12.5) | 1 ( 6.7) |  |  |
| Grade 2 | 35 (17.1) | 10 ( 17.5) | 2 ( 4.7) | 12 ( 23.5) | 5 ( 16.1) | 1 ( 12.5) | 5 ( 33.3) |  |  |
| Grade 3 | 10 ( 4.9) | 2 ( 3.5) | 3 ( 7.0) | 1 ( 2.0) | 0 ( 0.0) | 2 ( 25.0) | 2 ( 13.3) |  |  |
| Grade 4 | 40 (19.5) | 12 ( 21.1) | 9 ( 20.9) | 13 ( 25.5) | 2 ( 6.5) | 2 ( 25.0) | 2 ( 13.3) |  |  |
| Grade 5 | 65 (31.7) | 11 ( 19.3) | 13 ( 30.2) | 18 ( 35.3) | 16 ( 51.6) | 2 ( 25.0) | 5 ( 33.3) |  |  |
| Modified Fisher scale |  |  |  |  |  |  |  | 0.672 | 0.009 |
| 0 | 3 ( 1.5) | 3 ( 5.3) | 0 ( 0.0) | 0 ( 0.0) | 0 ( 0.0) | 0 ( 0.0) | 0 ( 0.0) |  |  |
| 1 | 23 (11.2) | 12 ( 21.1) | 2 ( 4.7) | 1 ( 2.0) | 5 ( 16.1) | 0 ( 0.0) | 3 ( 20.0) |  |  |
| 2 | 11 ( 5.4) | 6 ( 10.5) | 3 ( 7.0) | 1 ( 2.0) | 0 ( 0.0) | 0 ( 0.0) | 1 ( 6.7) |  |  |
| 3 | 65 (31.7) | 12 ( 21.1) | 19 ( 44.2) | 15 ( 29.4) | 13 ( 41.9) | 2 ( 25.0) | 4 ( 26.7) |  |  |
| 4 | 103 (50.2) | 24 ( 42.1) | 19 ( 44.2) | 34 ( 66.7) | 13 ( 41.9) | 6 ( 75.0) | 7 ( 46.7) |  |  |
| Intracerebral hemorrhage | 44 (21.7) | 14 ( 25.0) | 7 ( 16.3) | 14 ( 27.5) | 7 ( 22.6) | 1 ( 12.5) | 1 ( 7.1) | 0.262 | 0.513 |
| Acute hydrocephalus |  |  |  |  |  |  |  | 0.546 | 0.062 |
| Indeterminate | 15 ( 7.4) | 3 ( 5.5) | 4 ( 9.3) | 7 ( 13.7) | 1 ( 3.2) | 0 ( 0.0) | 0 ( 0.0) |  |  |
| No | 121 (59.6) | 35 ( 63.6) | 29 ( 67.4) | 30 ( 58.8) | 13 ( 41.9) | 3 ( 37.5) | 11 ( 73.3) |  |  |
| Yes | 67 (33.0) | 17 ( 30.9) | 10 ( 23.3) | 14 ( 27.5) | 17 ( 54.8) | 5 ( 62.5) | 4 ( 26.7) |  |  |
| Aneurysm location |  |  |  |  |  |  |  | 0.495 | 0.367 |
| Anterior communicating | 51 (25.0) | 14 ( 24.6) | 16 ( 37.2) | 11 ( 21.6) | 6 ( 19.4) | 1 ( 12.5) | 3 ( 21.4) |  |  |
| Middle cerebral artery bifurcation | 50 (24.5) | 19 ( 33.3) | 7 ( 16.3) | 12 ( 23.5) | 8 ( 25.8) | 2 ( 25.0) | 2 ( 14.3) |  |  |
| Posterior communicating | 42 (20.6) | 9 ( 15.8) | 8 ( 18.6) | 12 ( 23.5) | 10 ( 32.3) | 2 ( 25.0) | 1 ( 7.1) |  |  |
| Other | 61 (29.9) | 15 ( 26.3) | 12 ( 27.9) | 16 ( 31.4) | 7 ( 22.6) | 3 ( 37.5) | 8 ( 57.1) |  |  |
| Aneurysm diameter | 5.3 [3.8, 7.9] | 5.7 [4.1, 8.2] | 4.2 [3.1, 6.7] | 5.1 [3.8, 8.4] | 6.3 [4.6, 8.6] | 4.2 [3.5, 6.0] | 4.4 [4.0, 5.5] | 0.416 | 0.124 |
| Aneurysm pathology |  |  |  |  |  |  |  | 0.479 | 0.053 |
| Dissecting | 10 ( 5.1) | 0 ( 0.0) | 4 ( 9.3) | 1 ( 2.0) | 3 ( 10.0) | 1 ( 14.3) | 1 ( 7.7) |  |  |
| Fusiform | 15 ( 7.6) | 1 ( 1.9) | 5 ( 11.6) | 3 ( 6.0) | 5 ( 16.7) | 1 ( 14.3) | 0 ( 0.0) |  |  |
| Saccular | 172 (87.3) | 53 ( 98.1) | 34 ( 79.1) | 46 ( 92.0) | 22 ( 73.3) | 5 ( 71.4) | 12 ( 92.3) |  |  |
| Aneurysm treatment: open surgery | 30 (14.6) | 4 ( 7.0) | 7 ( 16.3) | 1 ( 2.0) | 11 ( 35.5) | 0 ( 0.0) | 7 ( 46.7) | 0.661 | <0.001 |
| Time from onset to open surgery | 12.6 [5.7, 42.9] | 10.3 [7.4, 59.1] | 4.7 [4.4, 10.5] | 396.6 [201.3, 591.9] | 26.5 [15.6, 136.1] | - | 7.0 [5.4, 13.1] | NA | 0.055 |
| Time from arrival to open surgery | 6.0 [3.9, 13.7] | 4.7 [4.6, 5.0] | 3.9 [3.7, 6.0] | 366.4 [184.2, 548.6] | 14.5 [6.0, 79.7] | - | 6.6 [4.1, 11.3] | NA | 0.185 |
| Aneurysm treatment: endovascular | 152 (74.1) | 50 ( 87.7) | 29 ( 67.4) | 47 ( 92.2) | 14 ( 45.2) | 7 ( 87.5) | 5 ( 33.3) | 0.718 | <0.001 |
| Endovascular surgery method |  |  |  |  |  |  |  | 0.985 | <0.001 |
| Coil | 104 (61.2) | 42 ( 75.0) | 26 ( 83.9) | 16 ( 33.3) | 12 ( 70.6) | 5 ( 62.5) | 3 ( 30.0) |  |  |
| Flow diverter | 2 ( 1.2) | 0 ( 0.0) | 0 ( 0.0) | 1 ( 2.1) | 0 ( 0.0) | 1 ( 12.5) | 0 ( 0.0) |  |  |
| Stent only | 2 ( 1.2) | 0 ( 0.0) | 2 ( 6.5) | 0 ( 0.0) | 0 ( 0.0) | 0 ( 0.0) | 0 ( 0.0) |  |  |
| Stent/coil | 47 (27.6) | 9 ( 16.1) | 3 ( 9.7) | 29 ( 60.4) | 2 ( 11.8) | 1 ( 12.5) | 3 ( 30.0) |  |  |
| Vessel occlusion | 1 ( 0.6) | 0 ( 0.0) | 0 ( 0.0) | 1 ( 2.1) | 0 ( 0.0) | 0 ( 0.0) | 0 ( 0.0) |  |  |
| Time from onset to endovascular surgery, h | 6.5 [4.0, 19.3] | 6.6 [4.3, 16.4] | 7.2 [3.2, 26.4] | 4.6 [3.7, 8.9] | 13.1 [7.5, 33.8] | 11.8 [5.6, 16.9] | 7.0 [4.3, 13.6] | 0.332 | 0.265 |
| Time from arrival to endovascular surgery, h | 2.7 [2.0, 5.0] | 2.8 [2.3, 3.8] | 3.3 [2.3, 4.9] | 1.9 [1.5, 2.5] | 11.1 [5.3, 21.6] | 5.5 [4.0, 11.2] | 6.6 [3.2, 8.9] | 0.435 | <0.001 |
| EVD | 63 (30.7) | 11 ( 19.3) | 11 ( 25.6) | 23 ( 45.1) | 9 ( 29.0) | 1 ( 12.5) | 8 ( 53.3) | 0.432 | 0.016 |
| Lumbar drainage | 62 (30.2) | 30 ( 52.6) | 16 ( 37.2) | 1 ( 2.0) | 8 ( 25.8) | 6 ( 75.0) | 1 ( 6.7) | 0.907 | <0.001 |
| Time from onset to EVD or lumbar drainage | 10.1 [6.5, 20.3] | 9.3 [7.0, 15.6] | 13.1 [5.3, 27.5] | 6.7 [5.5, 12.7] | 11.8 [7.1, 41.2] | 10.9 [9.8, 16.3] | 11.4 [7.5, 16.3] | 0.362 | 0.600 |
| Time from arrival to EVD or lumbar drainage | 5.9 [3.6, 11.3] | 5.6 [4.2, 8.2] | 6.6 [4.4, 22.6] | 3.5 [3.3, 7.0] | 7.4 [4.6, 14.7] | 7.3 [4.2, 14.8] | 11.0 [6.6, 14.8] | 0.144 | 0.098 |
| Vasospasm prophylaxis, nimodipine PO | 110 (53.9) | 48 ( 85.7) | 0 ( 0.0) | 36 ( 70.6) | 15 ( 48.4) | 3 ( 37.5) | 8 ( 53.3) | 0.996 | <0.001 |
| Vasospasm prophylaxis, nimodipine IV | 123 (60.3) | 6 ( 10.7) | 37 ( 86.0) | 38 ( 74.5) | 26 ( 83.9) | 5 ( 62.5) | 11 ( 73.3) | 0.786 | <0.001 |
| Vasospasm prophylaxis, magnesium IV | 58 (28.4) | 20 ( 35.7) | 37 ( 86.0) | 1 ( 2.0) | 0 ( 0.0) | 0 ( 0.0) | 0 ( 0.0) | 1.310 | <0.001 |
| Vasospasm prophylaxis, milrinone IV | 7 ( 3.4) | 0 ( 0.0) | 0 ( 0.0) | 2 ( 3.9) | 0 ( 0.0) | 3 ( 37.5) | 2 ( 13.3) | 0.509 | <0.001 |
| TCD vasospasm |  |  |  |  |  |  |  | 0.672 | 0.023 |
| No | 137 (71.0) | 36 ( 63.2) | 30 ( 69.8) | 40 ( 78.4) | 24 ( 82.8) | 3 ( 37.5) | 4 ( 80.0) |  |  |
| Not performed or untestable | 33 (17.1) | 15 ( 26.3) | 3 ( 7.0) | 8 ( 15.7) | 4 ( 13.8) | 3 ( 37.5) | 0 ( 0.0) |  |  |
| Yes | 23 (11.9) | 6 ( 10.5) | 10 ( 23.3) | 3 ( 5.9) | 1 ( 3.4) | 2 ( 25.0) | 1 ( 20.0) |  |  |
| Angiographic Vasospasm |  |  |  |  |  |  |  | 0.750 | <0.001 |
| DSA proven vasospasm | 14 ( 7.2) | 10 ( 17.5) | 1 ( 2.3) | 3 ( 5.9) | 0 ( 0.0) | 0 ( 0.0) | 0 ( 0.0) |  |  |
| Vasospasm suspected on CTA or MRA | 17 ( 8.8) | 1 ( 1.8) | 3 ( 7.0) | 5 ( 9.8) | 2 ( 6.7) | 4 ( 50.0) | 2 ( 40.0) |  |  |
| Endovascular vasospasm therapy | 17 (9.0) | 10 ( 17.5) | 3 ( 7.0) | 0 ( 0.0) | 1 ( 3.4) | 3 (42.9) | 0 (0.0) | 0.581 | <0.001 |
| Induced hypertension | 26 (13.7) | 12 ( 21.1) | 6 ( 14.0) | 2 ( 3.9) | 2 ( 6.9) | 3 ( 42.9) | 1 ( 33.3) | 0.495 | 0.016 |
| Neurologic deterioration | 51 (24.9) | 15 ( 26.3) | 9 ( 20.9) | 7 ( 13.7) | 14 ( 45.2) | 5 ( 62.5) | 1 ( 6.7) | 0.611 | 0.002 |
| Rebleeding | 19 ( 9.4) | 8 ( 14.3) | 3 ( 7.0) | 4 ( 7.8) | 3 ( 9.7) | 0 ( 0.0) | 1 ( 7.1) | 0.225 | 0.709 |
| Delayed cerebral ischemia | 27 (13.9) | 14 ( 24.6) | 6 ( 14.0) | 3 ( 5.9) | 1 ( 3.4) | 3 ( 37.5) | 0 ( 0.0) | 0.527 | 0.009 |
| Delayed cerebral ischemia, infarct | 18 ( 9.4) | 9 ( 16.4) | 3 ( 7.0) | 3 ( 5.9) | 1 ( 3.4) | 1 ( 12.5) | 1 ( 20.0) | 0.517 | 0.296 |
| Delayed hydrocephalus | 14 ( 6.8) | 1 ( 1.8) | 2 ( 4.7) | 2 ( 3.9) | 5 ( 16.1) | 4 ( 50.0) | 0 ( 0.0) | 0.597 | <0.001 |
| POLST | 29 (14.1) | 4 ( 7.0) | 4 ( 9.3) | 6 ( 11.8) | 10 ( 32.3) | 1 ( 12.5) | 4 ( 26.7) | 0.318 | 0.017 |
| Mortality during NCCU stay | 32 (15.6) | 7 ( 12.3) | 2 ( 4.7) | 11 ( 21.6) | 9 ( 29.0) | 1 ( 12.5) | 2 ( 13.3) | 0.283 | 0.072 |
| Length of NCCU stay, days | 8.0 [4.8, 15.5] | 5.9 [4.5, 13.5] | 9.3 [4.0, 16.0] | 8.8 [6.1, 12.9] | 10.3 [5.0, 19.7] | 13.5 [5.5, 17.0] | 8.0 [3.2, 12.6] | 0.217 | 0.310 |
| In-hospital mortality | 39 (19.0) | 8 ( 14.0) | 4 ( 9.3) | 13 ( 25.5) | 9 ( 29.0) | 1 ( 12.5) | 4 ( 26.7) | 0.265 | 0.171 |
| mRS at 6 months, 0 - 3 | 120 (60.0) | 39 ( 70.9) | 29 ( 69.0) | 25 ( 50.0) | 16 ( 51.6) | 2 ( 28.6) | 9 ( 60.0) | 0.392 | 0.074 |
| GOSE at 6 months, 5 - 8 | 112 (56.0) | 36 ( 65.5) | 25 ( 59.5) | 25 ( 50.0) | 16 ( 51.6) | 1 ( 14.3) | 9 ( 60.0) | 0.444 | 0.144 |
| Shunt dependency at 6 months |  |  |  |  |  |  |  | 0.935 | <0.001 |
| No | 172 (85.1) | 50 ( 90.9) | 36 ( 83.7) | 49 ( 98.0) | 22 ( 71.0) | 2 ( 25.0) | 13 ( 86.7) |  |  |
| Previous shunt (+) | 9 ( 4.5) | 0 ( 0.0) | 0 ( 0.0) | 1 ( 2.0) | 3 ( 9.7) | 5 ( 62.5) | 0 ( 0.0) |  |  |
| Yes | 21 (10.4) | 5 ( 9.1) | 7 ( 16.3) | 0 ( 0.0) | 6 ( 19.4) | 1 ( 12.5) | 2 ( 13.3) |  |  |

SMD: Standardized Mean Difference; GCS: Glasgow Coma Scale; SBP: Systolic Blood Pressure; DBP: Diastolic Blood Pressure; HR: Heart Rate; RR: Respiratory Rate; SpO2: Peripheral Oxygen Saturation; BT: Body Temperature; mRS: Modified Rankin Scale; ER, emergency room; WFNS: World Federation of Neurological Societies; EVD: External Ventricular Drain; TCD: Transcranial Doppler; DSA: Digital Subtraction Angiography; CTA: Computed Tomography Angiography; MRA: Magnetic Resonance Angiography; GOSE: Glasgow Outcome Scale Extended; NCCU: Neurocritical Care Unit; POLST: Physician Orders for Life-Sustaining Treatment.

**Supplemental Table 2. ICH Patient Characteristics Compared Across Participating Centers**

|  | Overall (n=478) | Hospital A (n=160,  33.5%) | Hospital B (n=92,  19.2%) | Hospital C (n=64,  13.4%) | Hospital D (n=80,  16.7%) | Hospital E (n=27,  5.6%) | Hospital F (n=55,  11.5%) | SMD | P-value |
| --- | --- | --- | --- | --- | --- | --- | --- | --- | --- |
| Age, years | 64.8 (16.8) | 67.3 (16.3) | 61.4 (18.7) | 64.8 (13.7) | 62.7 (18.3) | 63.0 (15.9) | 67.4 (15.6) | 0.184 | 0.070 |
| Female | 229 (47.9) | 73 ( 45.6) | 47 ( 51.1) | 31 ( 48.4) | 36 ( 45.0) | 13 ( 48.1) | 29 ( 52.7) | 0.074 | 0.913 |
| Hypertension | 257 (54.0) | 94 ( 58.8) | 46 ( 50.5) | 32 ( 50.0) | 43 ( 54.4) | 13 ( 48.1) | 29 ( 52.7) | 0.092 | 0.744 |
| Diabete mellitus | 120 (25.2) | 42 ( 26.2) | 24 ( 26.4) | 17 ( 26.6) | 20 ( 25.3) | 8 ( 29.6) | 9 ( 16.4) | 0.112 | 0.734 |
| Previous stroke | 89 (18.7) | 39 ( 24.5) | 16 ( 17.6) | 5 ( 7.8) | 14 ( 17.7) | 5 ( 18.5) | 10 ( 18.2) | 0.161 | 0.123 |
| Malignancy | 55 (11.6) | 14 ( 8.8) | 15 ( 16.5) | 4 ( 6.2) | 12 ( 15.2) | 6 ( 23.1) | 4 ( 7.3) | 0.235 | 0.062 |
| Chronic kidney disease | 36 ( 7.6) | 13 ( 8.2) | 9 ( 9.9) | 3 ( 4.7) | 5 ( 6.3) | 2 ( 7.4) | 4 ( 7.3) | 0.082 | 0.886 |
| Antiplatelet use | 102 (22.1) | 44 ( 27.5) | 18 ( 19.8) | 10 ( 20.0) | 17 ( 21.5) | 5 ( 18.5) | 8 ( 14.5) | 0.123 | 0.396 |
| Anticoagulant use | 71 (15.4) | 18 ( 11.2) | 10 ( 11.0) | 17 ( 35.4) | 16 ( 20.3) | 4 ( 14.8) | 6 ( 10.9) | 0.261 | 0.001 |
| Moderate-to-heavy alcohol use | 81 (16.9) | 30 ( 18.8) | 10 ( 10.9) | 23 ( 35.9) | 10 ( 12.5) | 6 ( 22.2) | 2 ( 3.6) | 0.434 | <0.001 |
| Current smoker | 82 (17.2) | 31 ( 19.4) | 12 ( 13.0) | 15 ( 23.4) | 11 ( 13.8) | 5 ( 18.5) | 8 ( 14.5) | 0.246 | 0.175 |
| Premorbid mRS ≥2 | 81 (16.9) | 43 ( 26.9) | 13 ( 14.1) | 0 ( 0.0) | 15 ( 18.8) | 7 ( 25.9) | 3 ( 5.5) | 0.621 | <0.001 |
| Mode of admission |  |  |  |  |  |  |  | 0.275 | 0.120 |
| Direct to ER | 358 (74.9) | 123 ( 76.9) | 69 ( 75.0) | 46 ( 71.9) | 57 ( 71.2) | 18 ( 66.7) | 45 ( 81.8) |  |  |
| From other hospital to ER | 19 ( 4.0) | 3 ( 1.9) | 2 ( 2.2) | 1 ( 1.6) | 7 ( 8.8) | 2 ( 7.4) | 4 ( 7.3) |  |  |
| In-hospital (except ER) | 101 (21.1) | 34 ( 21.2) | 21 ( 22.8) | 17 ( 26.6) | 16 ( 20.0) | 7 ( 25.9) | 6 ( 10.9) |  |  |
| Time from symptom onset to arrival, h | 1.7 [0.8, 5.5] | 1.5 [0.8, 5.5] | 1.7 [0.8, 4.7] | 1.9 [1.2, 3.1] | 2.2 [0.8, 9.4] | 4.2 [1.0, 8.4] | 1.4 [0.7, 3.4] | 0.167 | 0.339 |
| GCS score at arrival |  |  |  |  |  |  |  | 0.449 | <0.001 |
| 13 - 15 | 236 (49.6) | 104 ( 65.4) | 37 ( 40.7) | 23 ( 35.9) | 27 ( 33.8) | 14 ( 51.9) | 31 ( 56.4) |  | <0.001 |
| 9 - 12 | 98 (20.6) | 34 ( 21.4) | 19 ( 20.9) | 18 ( 28.1) | 17 ( 21.2) | 2 ( 7.4) | 8 ( 14.5) |  |  |
| 3 - 8 | 142 (29.8) | 21 ( 13.2) | 35 ( 38.5) | 23 ( 35.9) | 36 ( 45.0) | 11 ( 40.7) | 16 ( 29.1) |  |  |
| Median [IQR] | 12.0 [7.0, 15.0] | 14.0 [11.5, 15.0] | 10.0 [5.0, 14.0] | 11.0 [6.0, 13.0] | 9.0 [3.0, 14.0] | 13.0 [6.0, 14.5] | 13.0 [6.5, 15.0] | 0.361 |  |
| Pupillary response at arrival |  |  |  |  |  |  |  | 0.306 | 0.045 |
| neither one reactive | 101 (21.5) | 20 ( 12.8) | 26 ( 28.6) | 14 ( 22.2) | 18 ( 23.1) | 10 ( 37.0) | 13 ( 23.6) |  |  |
| one reactive | 23 ( 4.9) | 10 ( 6.4) | 3 ( 3.3) | 3 ( 4.8) | 3 ( 3.8) | 3 ( 11.1) | 1 ( 1.8) |  |  |
| both reactive | 346 (73.6) | 126 ( 80.8) | 62 ( 68.1) | 46 ( 73.0) | 57 ( 73.1) | 14 ( 51.9) | 41 ( 74.5) |  |  |
| Systolic blood pressure at arrival, mmHg | 167.1 (37.0) | 167.9 (34.9) | 174.1 (37.7) | 172.2 (38.0) | 161.7 (38.2) | 156.1 (38.2) | 160.6 (36.3) | 0.233 | 0.065 |
| Diastolic blood pressure at arrival, mmHg | 92.5 (22.6) | 91.9 (20.4) | 93.2 (23.0) | 100.6 (26.2) | 89.2 (21.4) | 88.6 (26.8) | 90.5 (21.4) | 0.205 | 0.043 |
| Heart rate at arrival, beats/min | 84.5 (19.1) | 83.9 (18.5) | 84.6 (20.4) | 82.5 (18.0) | 87.3 (19.0) | 89.3 (14.5) | 82.3 (21.8) | 0.184 | 0.410 |
| Respiratory rate at arrival, breaths/min | 19.4 (3.9) | 19.9 (3.5) | 20.6 (4.3) | 18.0 (2.3) | 18.2 (3.3) | 18.8 (5.2) | 19.7 (4.9) | 0.335 | <0.001 |
| Oxygen saturation at arrival, % | 96.9 (3.5) | 96.9 (2.2) | 97.0 (5.7) | 96.2 (2.8) | 97.0 (2.8) | 97.4 (2.2) | 97.0 (3.9) | 0.153 | 0.581 |
| Body temperature at arrival, °C | 36.5 (1.3) | 36.5 (0.7) | 36.4 (0.9) | 36.6 (1.1) | 36.8 (0.9) | 35.9 (4.1) | 36.6 (0.9) | 0.218 | 0.042 |
| ICH score |  |  |  |  |  |  |  | 0.545 | <0.001 |
| 0 | 90 (18.8) | 41 ( 25.6) | 9 ( 9.8) | 9 ( 14.1) | 14 ( 17.5) | 2 ( 7.4) | 15 ( 27.3) |  |  |
| 1 | 121 (25.3) | 49 ( 30.6) | 19 ( 20.7) | 21 ( 32.8) | 14 ( 17.5) | 8 ( 29.6) | 10 ( 18.2) |  |  |
| 2 | 103 (21.5) | 35 ( 21.9) | 16 ( 17.4) | 15 ( 23.4) | 20 ( 25.0) | 7 ( 25.9) | 10 ( 18.2) |  |  |
| 3 | 75 (15.7) | 20 ( 12.5) | 21 ( 22.8) | 10 ( 15.6) | 16 ( 20.0) | 3 ( 11.1) | 5 ( 9.1) |  |  |
| 4 | 65 (13.6) | 12 ( 7.5) | 22 ( 23.9) | 7 ( 10.9) | 12 ( 15.0) | 2 ( 7.4) | 10 ( 18.2) |  |  |
| 5 | 18 ( 3.8) | 2 ( 1.2) | 5 ( 5.4) | 2 ( 3.1) | 4 ( 5.0) | 4 ( 14.8) | 1 ( 1.8) |  |  |
| ICH location |  |  |  |  |  |  |  | 0.461 | 0.046 |
| Lobar | 181 (38.1) | 56 ( 35.0) | 37 ( 40.2) | 17 ( 26.6) | 41 ( 51.2) | 12 ( 44.4) | 18 ( 34.6) |  |  |
| Basal Ganglia | 145 (30.5) | 53 ( 33.1) | 33 ( 35.9) | 23 ( 35.9) | 18 ( 22.5) | 4 ( 14.8) | 14 ( 26.9) |  |  |
| Thalamus | 82 (17.3) | 32 ( 20.0) | 6 ( 6.5) | 14 ( 21.9) | 11 ( 13.8) | 6 ( 22.2) | 13 ( 25.0) |  |  |
| Cerebellum | 36 ( 7.6) | 9 ( 5.6) | 8 ( 8.7) | 3 ( 4.7) | 7 ( 8.8) | 3 ( 11.1) | 6 ( 11.5) |  |  |
| Brainstem | 31 ( 6.5) | 10 ( 6.2) | 8 ( 8.7) | 7 ( 10.9) | 3 ( 3.8) | 2 ( 7.4) | 1 ( 1.9) |  |  |
| ICH laterality |  |  |  |  |  |  |  | 0.248 | 0.878 |
| Left | 205 (43.2) | 69 ( 43.4) | 35 ( 38.0) | 28 ( 43.8) | 39 ( 48.8) | 8 ( 29.6) | 26 ( 50.0) |  |  |
| Right | 200 (42.2) | 69 ( 43.4) | 42 ( 45.7) | 27 ( 42.2) | 30 ( 37.5) | 14 ( 51.9) | 18 ( 34.6) |  |  |
| Infratentorial only | 65 (13.7) | 19 ( 11.9) | 15 ( 16.3) | 9 ( 14.1) | 10 ( 12.5) | 5 ( 18.5) | 7 ( 13.5) |  |  |
| Bilateral | 4 ( 0.8) | 2 ( 1.3) | 0 ( 0.0) | 0 ( 0.0) | 1 ( 1.2) | 0 ( 0.0) | 1 ( 1.9) |  |  |
| ICH volume |  |  |  |  |  |  |  | 0.348 | <0.001 |
| <30 | 298 (62.9) | 123 ( 76.9) | 42 ( 45.7) | 43 ( 67.2) | 41 ( 51.2) | 13 ( 48.1) | 36 ( 70.6) |  |  |
| ≥ 30, <60 | 96 (20.3) | 19 ( 11.9) | 27 ( 29.3) | 13 ( 20.3) | 21 ( 26.2) | 8 ( 29.6) | 8 ( 15.7) |  |  |
| ≥ 60 | 80 (16.9) | 18 ( 11.2) | 23 ( 25.0) | 8 ( 12.5) | 18 ( 22.5) | 6 ( 22.2) | 7 ( 13.7) |  |  |
| Median [IQR] | 18.1 [6.2, 43.9] | 9.8 [2.8, 26.5] | 31.6 [16.2, 59.2] | 17.9 [7.4, 39.8] | 28.0 [11.0, 58.0] | 31.0 [15.3, 49.1] | 12.0 [6.5, 34.0] | 0.325 | <0.001 |
| Intraventricular hemorrhage | 233 (48.7) | 72 ( 45.0) | 56 ( 60.9) | 34 ( 53.1) | 30 ( 37.5) | 15 ( 55.6) | 26 ( 47.3) | 0.209 | 0.044 |
| Modifed Graeb score | 13.0 [6.0, 19.0] | 13.0 [7.0, 17.8] | 15.0 [7.5, 22.5] | 11.0 [5.8, 18.2] | 14.0 [7.0, 21.5] | 11.0 [10.0, 19.0] | 12.5 [4.5, 18.5] | 0.167 | 0.526 |
| Acute hydrocephalus | 102 (21.3) | 37 ( 23.1) | 24 ( 26.1) | 8 ( 12.5) | 19 ( 23.8) | 6 ( 22.2) | 8 ( 14.5) | 0.165 | 0.276 |
| Midline shift, mm | 8.0 [5.0, 12.0] | 6.5 [4.3, 11.9] | 8.8 [5.8, 11.9] | 6.5 [4.1, 12.0] | 8.0 [5.0, 10.0] | 9.2 [6.2, 13.0] | 9.5 [6.8, 16.5] | 0.336 | 0.127 |
| Cisternal compression | 96 (20.3) | 15 ( 9.4) | 31 ( 33.7) | 7 ( 10.9) | 19 ( 23.8) | 7 ( 25.9) | 17 ( 34.7) | 0.330 | <0.001 |
| 4th ventricle effacement | 53 (11.3) | 16 ( 10.0) | 6 ( 6.5) | 4 ( 6.2) | 8 ( 10.0) | 4 ( 14.8) | 15 ( 31.2) | 0.281 | <0.001 |
| ICH cause |  |  |  |  |  |  |  | 0.468 | 0.005 |
| Arteriovenous malformation | 25 ( 5.3) | 5 ( 3.1) | 7 ( 7.6) | 2 ( 3.1) | 7 ( 8.8) | 3 ( 11.1) | 1 ( 2.0) |  |  |
| Moyamoya disease | 19 ( 4.0) | 3 ( 1.9) | 7 ( 7.6) | 3 ( 4.7) | 2 ( 2.5) | 2 ( 7.4) | 2 ( 3.9) |  |  |
| Others, specify | 15 ( 3.2) | 4 ( 2.5) | 2 ( 2.2) | 3 ( 4.7) | 1 ( 1.2) | 2 ( 7.4) | 3 ( 5.9) |  |  |
| Ruptured aneurysm | 1 ( 0.2) | 0 ( 0.0) | 1 ( 1.1) | 0 ( 0.0) | 0 ( 0.0) | 0 ( 0.0) | 0 ( 0.0) |  |  |
| Spontaneous | 402 (84.8) | 148 ( 92.5) | 68 ( 73.9) | 56 ( 87.5) | 68 ( 85.0) | 18 ( 66.7) | 44 ( 86.3) |  |  |
| Thrombolytic agent (rt-PA) | 1 ( 0.2) | 0 ( 0.0) | 1 ( 1.1) | 0 ( 0.0) | 0 ( 0.0) | 0 ( 0.0) | 0 ( 0.0) |  |  |
| Trauma | 2 ( 0.4) | 0 ( 0.0) | 0 ( 0.0) | 0 ( 0.0) | 2 ( 2.5) | 0 ( 0.0) | 0 ( 0.0) |  |  |
| Tumor | 9 ( 1.9) | 0 ( 0.0) | 6 ( 6.5) | 0 ( 0.0) | 0 ( 0.0) | 2 ( 7.4) | 1 ( 2.0) |  |  |
| ICH surgery | 178 (37.2) | 53 ( 33.1) | 39 ( 42.4) | 17 ( 26.6) | 35 ( 43.8) | 18 ( 66.7) | 16 ( 29.1) | 0.360 | 0.003 |
| Time from onset to surgery | 5.7 [3.8, 13.4] | 5.5 [3.5, 7.3] | 4.4 [3.5, 7.3] | 5.0 [3.7, 8.5] | 10.9 [5.5, 24.8] | 24.0 [5.4, 78.1] | 6.5 [4.0, 24.4] | 0.437 | 0.001 |
| Time from arrival to surgery | 3.5 [2.4, 8.1] | 3.7 [2.6, 4.8] | 2.5 [1.9, 3.4] | 2.5 [2.0, 3.0] | 8.1 [3.7, 16.0] | 6.9 [2.2, 46.3] | 4.2 [2.9, 21.4] | 0.395 | <0.001 |
| - EVD | 71 (14.9) | 17 ( 10.6) | 13 ( 14.1) | 11 ( 17.2) | 12 ( 15.0) | 6 ( 22.2) | 12 ( 21.8) | 0.150 | 0.322 |
| - DC + HE | 44 ( 9.2) | 9 ( 5.6) | 17 ( 18.5) | 3 ( 4.7) | 7 ( 8.8) | 5 ( 18.5) | 3 ( 5.5) | 0.239 | 0.004 |
| - Craniotomy + HE | 49 (10.3) | 16 ( 10.0) | 9 ( 9.8) | 0 ( 0.0) | 14 ( 17.5) | 6 ( 22.2) | 4 ( 7.3) | 0.328 | 0.005 |
| - DC only | 4 ( 0.8) | 0 ( 0.0) | 0 ( 0.0) | 0 ( 0.0) | 1 ( 1.2) | 0 ( 0.0) | 3 ( 5.5) | 0.149 | 0.004 |
| - Minimally invasive surgery | 31 ( 6.5) | 16 ( 10.0) | 1 ( 1.1) | 8 ( 12.5) | 5 ( 6.2) | 1 ( 3.7) | 0 ( 0.0) | 0.282 | 0.008 |
| Neurologic deterioration | 72 (16.0) | 19 ( 14.3) | 13 ( 14.1) | 7 ( 10.9) | 17 ( 21.2) | 7 ( 26.9) | 9 ( 16.4) | 0.180 | 0.336 |
| Hematoma expansion | 36 ( 8.3) | 12 ( 9.0) | 6 ( 6.9) | 6 ( 9.5) | 4 ( 5.2) | 1 ( 4.3) | 7 ( 13.7) | 0.150 | 0.571 |
| Hematoma expansion, treatment |  |  |  |  |  |  |  | 1.273 | 0.381 |
| Any surgical therapy | 15 (39.5) | 5 ( 41.7) | 1 ( 16.7) | 2 ( 33.3) | 2 ( 28.6) | 1 (100.0) | 4 ( 66.7) |  |  |
| Medical therapy only | 14 (36.8) | 5 ( 41.7) | 2 ( 33.3) | 4 ( 66.7) | 2 ( 28.6) | 0 ( 0.0) | 1 ( 16.7) |  |  |
| None | 9 (23.7) | 2 ( 16.7) | 3 ( 50.0) | 0 ( 0.0) | 3 ( 42.9) | 0 ( 0.0) | 1 ( 16.7) |  |  |
| POLST | 61 (12.8) | 8 ( 5.1) | 15 ( 16.3) | 8 ( 12.5) | 17 ( 21.2) | 4 ( 14.8) | 9 ( 16.4) | 0.190 | 0.010 |
| Mortality during NCCU stay | 72 (15.1) | 14 ( 8.8) | 20 ( 21.7) | 14 ( 21.9) | 14 ( 17.5) | 4 ( 14.8) | 6 ( 10.9) | 0.188 | 0.040 |
| Length of NCCU stay, days | 5.5 [2.7, 13.5] | 4.9 [2.8, 12.5] | 5.6 [2.8, 12.4] | 5.4 [2.1, 13.8] | 7.0 [3.0, 14.7] | 4.9 [1.7, 13.8] | 4.9 [2.3, 15.8] | 0.124 | 0.733 |
| In-hospital mortality | 84 (17.6) | 15 ( 9.5) | 22 ( 23.9) | 14 ( 21.9) | 18 ( 22.5) | 5 ( 18.5) | 10 ( 18.2) | 0.158 | 0.037 |
| mRS at 6 months, 0 - 3 | 197 (42.6) | 77 ( 48.4) | 37 ( 41.6) | 18 ( 29.0) | 32 ( 40.0) | 8 ( 36.4) | 25 ( 50.0) | 0.197 | 0.126 |
| GOSE at 6 months, 5 - 8 | 168 (36.4) | 62 ( 39.0) | 26 ( 29.2) | 16 ( 25.8) | 31 ( 38.8) | 8 ( 36.4) | 25 ( 50.0) | 0.215 | 0.086 |

SMD: Standardized Mean Difference; ICH: Intracerebral Hemorrhage; mRS: Modified Rankin Scale; ER, emergency room; GCS: Glasgow Coma Scale; IQR: Interquartile Range; EVD: External Ventricular Drain; DC: Decompressive Craniectomy; HE: Hematoma Evacuation; NCCU: Neurocritical Care Unit; GOSE: Glasgow Outcome Scale Extended; POLST: Physician Orders for Life-Sustaining Treatment.

**Supplemental Table 3. TBI Patient Characteristics Compared Across Participating Centers**

|  | Overall (n=442) | Hospital A  (n=74,  16.8%) | Hospital B  (n=108,  24.5%) | Hospital C  (n=77,  17.5%) | Hospital D  (n=84,  19.0%) | Hospital E  (n=29,  6.6%) | Hospital F  (n=69,  15.6%) | SMD | P-value |
| --- | --- | --- | --- | --- | --- | --- | --- | --- | --- |
| Age, years | 68.1 (17.1) | 68.8 (16.7) | 68.1 (18.3) | 67.1 (16.8) | 67.5 (18.1) | 69.6 (12.6) | 69.2 (15.9) | 0.074 | 0.965 |
| Female | 141 (31.9) | 27 ( 36.5) | 34 ( 31.5) | 25 ( 32.5) | 21 ( 25.0) | 13 ( 44.8) | 20 ( 29.0) | 0.174 | 0.405 |
| Hypertension | 243 (55.1) | 41 ( 55.4) | 57 ( 52.8) | 45 ( 58.4) | 50 ( 59.5) | 14 ( 50.0) | 36 ( 52.2) | 0.093 | 0.878 |
| Diabete mellitus | 145 (33.0) | 31 ( 41.9) | 35 ( 32.4) | 19 ( 24.7) | 30 ( 35.7) | 9 ( 33.3) | 21 ( 30.4) | 0.147 | 0.351 |
| Previous stroke | 79 (17.9) | 17 ( 23.0) | 27 ( 25.0) | 5 ( 6.5) | 17 ( 20.2) | 5 ( 17.9) | 8 ( 11.6) | 0.241 | 0.016 |
| Malignancy | 70 (15.9) | 5 ( 6.8) | 16 ( 14.8) | 4 ( 5.2) | 23 ( 27.4) | 9 ( 34.6) | 13 ( 18.8) | 0.386 | <0.001 |
| Chronic kidney disease | 44 (10.0) | 6 ( 8.1) | 11 ( 10.2) | 3 ( 3.9) | 9 ( 10.7) | 11 ( 39.3) | 4 ( 5.8) | 0.361 | <0.001 |
| Antiplatelet use | 118 (27.7) | 26 ( 35.1) | 27 ( 25.0) | 16 ( 25.8) | 24 ( 28.6) | 9 ( 32.1) | 16 ( 23.2) | 0.124 | 0.616 |
| Anticoagulant use | 68 (15.8) | 7 ( 9.5) | 15 ( 13.9) | 26 ( 38.8) | 9 ( 10.7) | 5 ( 17.9) | 6 ( 8.7) | 0.307 | <0.001 |
| Moderate-to-heavy alcohol use | 72 (16.3) | 16 ( 21.6) | 13 ( 12.0) | 25 ( 32.5) | 9 ( 10.7) | 4 ( 13.8) | 4 ( 5.8) | 0.458 | <0.001 |
| Current smoker | 97 (21.9) | 20 ( 27.0) | 21 ( 19.4) | 18 ( 23.4) | 17 ( 20.2) | 6 ( 20.7) | 15 ( 21.7) | 0.277 | 0.123 |
| Premorbid mRS ≥2 | 76 (17.2) | 19 ( 25.7) | 17 ( 15.7) | 4 ( 5.2) | 16 ( 19.0) | 14 ( 48.3) | 6 ( 8.7) | 0.619 | <0.001 |
| Mode of admission |  |  |  |  |  |  |  | 0.385 | <0.001 |
| Direct to ER | 320 (72.4) | 56 ( 75.7) | 85 ( 78.7) | 43 ( 55.8) | 58 ( 69.0) | 20 ( 69.0) | 57 ( 82.6) |  |  |
| In-hospital (except ER) | 13 ( 2.9) | 1 ( 1.4) | 1 ( 0.9) | 0 ( 0.0) | 6 ( 7.1) | 3 ( 10.3) | 2 ( 2.9) |  |  |
| From other hospital to ER | 109 (24.7) | 17 ( 23.0) | 22 ( 20.4) | 34 ( 44.2) | 20 ( 23.8) | 6 ( 20.7) | 10 ( 14.5) |  |  |
| Time from symptom onset to arrival, h | 1.9 [0.8, 6.8] | 2.0 [0.9, 5.2] | 1.9 [0.9, 14.6] | 1.7 [0.8, 4.6] | 1.6 [0.5, 7.3] | 1.3 [1.0, 3.2] | 2.4 [0.7, 12.9] | 0.231 | 0.387 |
| GCS score at arrival |  |  |  |  |  |  |  | 0.461 | <0.001 |
| 13 - 15 | 244 (56.0) | 47 ( 63.5) | 78 ( 73.6) | 28 ( 38.4) | 33 ( 39.3) | 12 ( 41.4) | 45 ( 65.2) |  |  |
| 9 - 12 | 65 (14.9) | 15 ( 20.3) | 12 ( 11.3) | 14 ( 19.2) | 12 ( 14.3) | 5 ( 17.2) | 7 ( 10.1) |  |  |
| 3 - 8 | 127 (29.1) | 12 ( 16.2) | 16 ( 15.1) | 31 ( 42.5) | 39 ( 46.4) | 12 ( 41.4) | 17 ( 24.6) |  |  |
| Median [IQR] | 13.0 [7.0, 15.0] | 14.0 [10.0, 15.0] | 15.0 [12.0, 15.0] | 12.0 [6.0, 14.0] | 9.5 [3.0, 15.0] | 12.0 [6.0, 14.0] | 14.0 [9.0, 15.0] | 0.446 | <0.001 |
| Pupillary response at arrival |  |  |  |  |  |  |  | 0.353 | 0.021 |
| neither one reactive | 82 (19.0) | 10 ( 14.1) | 12 ( 11.5) | 20 ( 27.4) | 20 ( 23.8) | 9 ( 31.0) | 10 ( 14.5) |  |  |
| one reactive | 27 ( 6.3) | 7 ( 9.9) | 5 ( 4.8) | 3 ( 4.1) | 6 ( 7.1) | 4 ( 13.8) | 2 ( 2.9) |  |  |
| both reactive | 322 (74.7) | 54 ( 76.1) | 87 ( 83.7) | 50 ( 68.5) | 58 ( 69.0) | 16 ( 55.2) | 57 ( 82.6) |  |  |
| Systolic blood pressure at arrival, mmHg | 145.3 (31.1) | 144.4 (27.6) | 147.7 (29.7) | 147.2 (36.4) | 146.7 (29.7) | 142.6 (41.0) | 139.8 (27.5) | 0.118 | 0.614 |
| Diastolic blood pressure at arrival, mmHg | 81.7 (18.8) | 79.9 (17.6) | 80.3 (18.8) | 90.4 (22.3) | 81.1 (16.4) | 77.8 (16.8) | 78.2 (16.9) | 0.249 | 0.001 |
| Heart rate at arrival, beats/min | 86.0 (21.3) | 88.0 (23.3) | 85.1 (21.3) | 85.5 (21.2) | 86.6 (19.9) | 89.7 (22.1) | 83.9 (21.0) | 0.121 | 0.771 |
| Respiratory rate at arrival, breaths/min | 19.2 (3.7) | 21.0 (4.2) | 19.4 (4.2) | 18.5 (2.9) | 17.9 (3.3) | 20.2 (3.4) | 19.3 (2.9) | 0.397 | <0.001 |
| Oxygen saturation at arrival, % | 97.0 (3.0) | 97.1 (2.2) | 98.0 (1.8) | 96.3 (3.5) | 96.5 (3.9) | 96.6 (3.8) | 97.1 (3.0) | 0.248 | 0.002 |
| Body temperature at arrival, °C | 36.5 (1.2) | 36.6 (0.7) | 36.6 (0.7) | 36.6 (0.9) | 36.2 (2.4) | 36.4 (0.8) | 36.6 (0.7) | 0.131 | 0.283 |
| Trauma, type |  |  |  |  |  |  |  | 0.580 | <0.001 |
| Fall from height > 1 meter (추락) | 39 ( 8.8) | 8 ( 10.8) | 12 ( 11.1) | 6 ( 7.8) | 4 ( 4.8) | 3 ( 10.3) | 6 ( 8.7) |  |  |
| Ground level fall | 281 (63.6) | 44 ( 59.5) | 81 ( 75.0) | 38 ( 49.4) | 63 ( 75.0) | 16 ( 55.2) | 39 ( 56.5) |  |  |
| Others | 16 ( 3.6) | 0 ( 0.0) | 3 ( 2.8) | 4 ( 5.2) | 0 ( 0.0) | 2 ( 6.9) | 7 ( 10.1) |  |  |
| Road traffic accident | 53 (12.0) | 14 ( 18.9) | 6 ( 5.6) | 11 ( 14.3) | 11 ( 13.1) | 6 ( 20.7) | 4 ( 5.8) |  |  |
| Unknown | 47 (10.6) | 6 ( 8.1) | 5 ( 4.6) | 16 ( 20.8) | 6 ( 7.1) | 1 ( 3.4) | 13 ( 18.8) |  |  |
| Violence/Assault | 6 ( 1.4) | 2 ( 2.7) | 1 ( 0.9) | 2 ( 2.6) | 0 ( 0.0) | 1 ( 3.4) | 0 ( 0.0) |  |  |
| Total ISS score | 20.2 (8.0) | 21.0 (5.6) | 18.3 (5.3) | 17.0 (10.4) | 22.7 (6.1) | 24.3 (14.5) | 20.5 (6.3) | 0.398 | <0.001 |
| Head/neck score ≥3 | 419 (94.8) | 73 ( 98.6) | 107 ( 99.1) | 62 ( 80.5) | 84 (100.0) | 24 ( 82.8) | 68 ( 98.6) | 0.372 | <0.001 |
| Face score ≥3 | 49 (11.1) | 11 ( 14.9) | 4 ( 3.7) | 7 ( 9.1) | 8 ( 9.5) | 10 ( 34.5) | 8 ( 11.6) | 0.328 | <0.001 |
| Chest score ≥3 | 29 ( 6.6) | 5 ( 6.8) | 8 ( 7.4) | 3 ( 3.9) | 4 ( 4.8) | 5 ( 17.2) | 3 ( 4.3) | 0.182 | 0.179 |
| Abdomen score ≥3 | 12 ( 2.7) | 0 ( 0.0) | 0 ( 0.0) | 2 ( 2.6) | 3 ( 3.6) | 4 ( 13.8) | 2 ( 2.9) | 0.262 | 0.001 |
| Extremities score ≥3 | 28 ( 6.3) | 7 ( 9.5) | 3 ( 2.8) | 5 ( 6.5) | 3 ( 3.6) | 9 ( 31.0) | 1 ( 1.5) | 0.368 | <0.001 |
| External score ≥3 | 17 ( 3.8) | 1 ( 1.4) | 0 ( 0.0) | 2 ( 2.6) | 2 ( 2.4) | 7 ( 24.1) | 5 ( 7.2) | 0.352 | <0.001 |
| Rotterdam CT score |  |  |  |  |  |  |  | 0.490 | 0.027 |
| 1 | 10 ( 2.3) | 1 ( 1.4) | 3 ( 2.8) | 3 ( 3.9) | 0 ( 0.0) | 0 ( 0.0) | 3 ( 4.3) |  |  |
| 2 | 107 (24.2) | 21 ( 28.4) | 31 ( 28.7) | 16 ( 20.8) | 20 ( 23.8) | 9 ( 31.0) | 10 ( 14.5) |  |  |
| 3 | 174 (39.4) | 31 ( 41.9) | 36 ( 33.3) | 31 ( 40.3) | 34 ( 40.5) | 11 ( 37.9) | 31 ( 44.9) |  |  |
| 4 | 90 (20.4) | 19 ( 25.7) | 27 ( 25.0) | 12 ( 15.6) | 14 ( 16.7) | 4 ( 13.8) | 13 ( 18.8) |  |  |
| 5 | 43 ( 9.7) | 2 ( 2.7) | 8 ( 7.4) | 11 ( 14.3) | 9 ( 10.7) | 3 ( 10.3) | 10 ( 14.5) |  |  |
| 6 | 16 ( 3.6) | 0 ( 0.0) | 3 ( 2.8) | 4 ( 5.2) | 7 ( 8.3) | 2 ( 6.9) | 0 ( 0.0) |  |  |
| Epidural hematoma | 66 (15.0) | 11 ( 14.9) | 10 ( 9.3) | 15 ( 19.5) | 11 ( 13.1) | 12 ( 41.4) | 7 ( 10.4) | 0.318 | 0.001 |
| Acute subdural hematoma | 308 (70.0) | 59 ( 79.7) | 63 ( 58.3) | 65 ( 84.4) | 53 ( 63.1) | 25 ( 86.2) | 42 ( 62.7) | 0.343 | <0.001 |
| Subacute/chronic subdural hematoma | 126 (28.6) | 22 ( 29.7) | 32 ( 29.6) | 16 ( 20.8) | 23 ( 27.4) | 11 ( 37.9) | 22 ( 32.8) | 0.151 | 0.509 |
| Subarachnoid hemorrhage | 246 (56.0) | 41 ( 55.4) | 51 ( 47.2) | 46 ( 59.7) | 44 ( 52.4) | 23 ( 79.3) | 41 ( 62.1) | 0.277 | 0.040 |
| Skull fracture | 142 (32.4) | 29 ( 39.2) | 33 ( 30.6) | 29 ( 37.7) | 24 ( 28.6) | 15 ( 51.7) | 12 ( 18.5) | 0.298 | 0.016 |
| Contusion | 155 (35.2) | 34 ( 45.9) | 32 ( 29.6) | 32 ( 41.6) | 21 ( 25.0) | 12 ( 41.4) | 24 ( 35.8) | 0.206 | 0.054 |
| Intracerebral hemorrhage | 109 (24.8) | 18 ( 24.3) | 36 ( 33.3) | 13 ( 16.9) | 16 ( 19.0) | 13 ( 44.8) | 13 ( 19.4) | 0.284 | 0.008 |
| Intraventricular hemorrhage | 42 ( 9.5) | 10 ( 13.5) | 5 ( 4.6) | 12 ( 15.6) | 7 ( 8.3) | 4 ( 13.8) | 4 ( 6.0) | 0.188 | 0.096 |
| Midline shift | 198 (45.3) | 32 ( 43.2) | 52 ( 48.1) | 37 ( 48.1) | 40 ( 47.6) | 12 ( 42.9) | 25 ( 38.5) | 0.092 | 0.822 |
| Median [IQR], mm | 9.7 (7.0) | 9.1 (5.6) | 10.3 (6.0) | 12.2 (8.2) | 9.4 (7.8) | 8.4 (7.5) | 8.1 (6.2) | 0.251 | 0.168 |
| Cistern compression |  |  |  |  |  |  |  | 0.305 | 0.047 |
| - Compressed | 104 (23.6) | 20 ( 27.0) | 23 ( 21.3) | 17 ( 22.1) | 14 ( 16.7) | 9 ( 31.0) | 20 ( 29.9) |  |  |
| - Absent | 42 ( 9.5) | 2 ( 2.7) | 8 ( 7.4) | 12 ( 15.6) | 14 ( 16.7) | 3 ( 10.3) | 3 ( 4.5) |  |  |
| 4th ventricle effacement | 19 ( 4.3) | 1 ( 1.4) | 2 ( 1.9) | 6 ( 7.8) | 6 ( 7.1) | 4 ( 13.8) | 0 ( 0.0) | 0.286 | 0.006 |
| Diffuse axonal injury | 31 ( 7.0) | 2 ( 2.7) | 2 ( 1.9) | 7 ( 9.1) | 6 ( 7.1) | 11 ( 37.9) | 3 ( 4.5) | 0.409 | <0.001 |
| Ischemia/infarct/hypoxic damage | 36 ( 8.2) | 4 ( 5.5) | 2 ( 1.9) | 2 ( 2.6) | 23 ( 27.4) | 2 ( 6.9) | 2 ( 3.0) | 0.315 | <0.001 |
| ICP monitoring |  |  |  |  |  |  |  | 0.521 | <0.001 |
| Parenchymal | 10 ( 2.3) | 3 ( 4.1) | 0 ( 0.0) | 0 ( 0.0) | 0 ( 0.0) | 7 ( 24.1) | 0 ( 0.0) |  |  |
| Ventricular | 9 ( 2.0) | 0 ( 0.0) | 4 ( 3.7) | 3 ( 3.9) | 1 ( 1.2) | 0 ( 0.0) | 1 ( 1.4) |  |  |
| Other | 6 ( 1.4) | 0 ( 0.0) | 0 ( 0.0) | 0 ( 0.0) | 1 ( 1.2) | 4 ( 13.8) | 1 ( 1.4) |  |  |
| Cranial surgery | 185 (42.0) | 34 ( 45.9) | 43 ( 39.8) | 25 ( 32.5) | 41 ( 48.8) | 17 ( 58.6) | 25 ( 36.2) | 0.239 | 0.094 |
| Decompressive craniectomy | 58 (13.1) | 12 ( 16.2) | 11 ( 10.2) | 9 ( 11.7) | 16 ( 19.0) | 4 ( 13.8) | 6 ( 8.7) | 0.141 | 0.375 |
| Craniotomy | 68 (15.4) | 18 ( 24.3) | 10 ( 9.3) | 12 ( 15.6) | 16 ( 19.0) | 6 ( 20.7) | 6 ( 8.7) | 0.215 | 0.042 |
| Burr-hole surgery | 67 (15.2) | 7 ( 9.5) | 23 ( 21.3) | 5 ( 6.5) | 15 ( 17.9) | 2 ( 6.9) | 15 ( 21.7) | 0.251 | 0.017 |
| EVD | 13 ( 2.9) | 1 ( 1.4) | 3 ( 2.8) | 2 ( 2.6) | 1 ( 1.2) | 4 ( 13.8) | 2 ( 2.9) | 0.193 | 0.019 |
| CRASH-3 trial eligible | 252 (57.0) | 42 ( 56.8) | 58 ( 53.7) | 51 ( 66.2) | 45 ( 53.6) | 20 ( 69.0) | 35 ( 50.7) | 0.182 | 0.273 |
| - TXA use | 150 (59.5) | 14 ( 33.3) | 23 ( 39.7) | 44 ( 86.3) | 40 ( 88.9) | 14 ( 70.0) | 15 ( 42.9) | 0.697 | <0.001 |
| Total TXA use | 226 (51.1) | 26 ( 35.1) | 33 ( 30.6) | 66 ( 85.7) | 66 ( 78.6) | 17 ( 58.6) | 18 ( 26.1) | 0.720 | <0.001 |
| - TXA use beyond CRASH-3 | 76 (33.6) | 12 (46.2) | 10 (30.3) | 22 (33.3) | 26 (39.4) | 3 (17.6) | 3 (16.7) | 0.324 | 0.203 |
| Time from onset to TXA use, h | 4.5 [2.2, 9.2] | 5.9 [3.4, 7.4] | 6.2 [3.2, 11.0] | 2.6 [1.8, 5.6] | 5.7 [3.0, 14.1] | 3.6 [1.2, 12.0] | 5.6 [2.4, 8.8] | 0.261 | 0.002 |
| Time from arrival to TXA, h | 1.5 [0.8, 4.2] | 1.7 [1.1, 2.5] | 2.9 [1.5, 7.8] | 0.8 [0.5, 1.0] | 2.2 [1.3, 5.7] | 1.0 [0.4, 9.5] | 3.0 [1.7, 7.5] | 0.365 | <0.001 |
| Neurologic deterioration | 107 (24.2) | 17 ( 23.0) | 21 ( 19.4) | 17 ( 22.1) | 36 ( 42.9) | 10 ( 34.5) | 6 ( 8.7) | 0.349 | <0.001 |
| - Hematoma expansion | 25 ( 5.7) | 6 ( 8.1) | 5 ( 4.6) | 10 ( 13.0) | 1 ( 1.2) | 2 ( 6.9) | 1 ( 1.4) | 0.234 | 0.014 |
| - Edema | 13 ( 2.9) | 3 ( 4.1) | 4 ( 3.7) | 1 ( 1.3) | 4 ( 4.8) | 1 ( 3.4) | 0 ( 0.0) | 0.147 | 0.500 |
| - Hydrocephalus | 4 ( 0.9) | 0 ( 0.0) | 1 ( 0.9) | 0 ( 0.0) | 2 ( 2.4) | 1 ( 3.4) | 0 ( 0.0) | 0.148 | 0.294 |
| - Infarct | 11 ( 2.5) | 2 ( 2.7) | 0 ( 0.0) | 2 ( 2.6) | 5 ( 6.0) | 1 ( 3.4) | 1 ( 1.4) | 0.156 | 0.197 |
| - Seizure | 21 ( 4.8) | 1 ( 1.4) | 6 ( 5.6) | 2 ( 2.6) | 10 ( 11.9) | 1 ( 3.4) | 1 ( 1.4) | 0.199 | 0.015 |
| - Hemorrhage | 10 ( 2.3) | 2 ( 2.7) | 4 ( 3.7) | 0 ( 0.0) | 4 ( 4.8) | 0 ( 0.0) | 0 ( 0.0) | 0.180 | 0.187 |
| - Medical complication | 25 ( 5.7) | 2 ( 2.7) | 0 ( 0.0) | 1 ( 1.3) | 20 ( 23.8) | 2 ( 6.9) | 0 ( 0.0) | 0.373 | <0.001 |
| - Other causes | 27 ( 6.1) | 2 ( 2.7) | 6 ( 5.6) | 3 ( 3.9) | 11 ( 13.1) | 2 ( 6.9) | 3 ( 4.3) | 0.164 | 0.083 |
| Mortality during NCCU stay | 63 (14.4) | 6 ( 8.2) | 15 ( 13.9) | 18 ( 23.4) | 13 ( 15.7) | 5 ( 17.2) | 6 ( 8.7) | 0.197 | 0.092 |
| Length of NCCU stay, days | 4.0 [1.7, 12.0] | 3.5 [2.4, 10.8] | 1.9 [0.9, 6.9] | 4.5 [1.8, 10.0] | 6.9 [3.7, 19.9] | 13.9 [6.7, 25.3] | 1.9 [1.1, 6.0] | 0.381 | <0.001 |
| In-hospital mortality | 78 (17.8) | 8 ( 11.0) | 16 ( 14.8) | 19 ( 24.7) | 17 ( 20.5) | 8 ( 27.6) | 10 ( 14.5) | 0.205 | 0.140 |
| POLST | 58 (13.2) | 8 ( 11.0) | 12 ( 11.1) | 5 ( 6.5) | 15 ( 18.1) | 9 ( 31.0) | 9 ( 13.0) | 0.266 | 0.019 |
| mRS at 6 months, 0 - 3 | 218 (51.1) | 38 ( 52.8) | 64 ( 59.3) | 34 ( 46.6) | 40 ( 47.6) | 5 ( 17.9) | 37 ( 59.7) | 0.368 | 0.003 |
| GOSE at 6 months, 5 - 8 | 187 (43.8) | 33 ( 45.8) | 51 ( 47.2) | 28 ( 38.4) | 37 ( 44.0) | 3 ( 10.7) | 35 ( 56.5) | 0.394 | 0.003 |

**Supplemental Table 4. Full Results of Fixed and Linear Mixed-Effects Models for utility-weighted modified Rankin Scale at 6 months**

|  | **Fixed effect model** | | | | **Mixed effect model** | | | |
| --- | --- | --- | --- | --- | --- | --- | --- | --- |
| **Term** | **Beta** | **CI lower** | **CI upper** | **P-value** | **Beta** | **CI lower** | **CI upper** | **P-value** |
| (Intercept) | -0.725 | -1.563 | 0.114 | 0.091 | -0.738 | -1.578 | 0.102 | 0.085 |
| Age | -0.005 | -0.006 | -0.004 | <0.001 | -0.005 | -0.006 | -0.004 | <0.001 |
| Type of ABI: ICH | Ref |  |  |  | Ref |  |  |  |
| Type of ABI: SAH | 0.099 | 0.046 | 0.152 | <0.001 | 0.101 | 0.048 | 0.154 | <0.001 |
| Type of ABI: TBI | 0.053 | 0.01 | 0.096 | 0.017 | 0.054 | 0.01 | 0.097 | 0.015 |
| Mode of admission: Direct | Ref |  |  |  | Ref |  |  |  |
| Mode of admission: In-hospital | -0.051 | -0.169 | 0.067 | 0.395 | -0.056 | -0.174 | 0.061 | 0.348 |
| Mode of admission: Other hospital | 0.043 | -0.001 | 0.087 | 0.053 | 0.044 | 0 | 0.088 | 0.052 |
| Onset to arrival time, h | 0 | 0 | 0.001 | 0.038 | 0 | 0 | 0.001 | 0.038 |
| GCS score at arrival | 0.033 | 0.028 | 0.039 | <0.001 | 0.034 | 0.028 | 0.04 | <0.001 |
| Pupil at arrival: both reactive | Ref |  |  |  | Ref |  |  |  |
| Pupil at arrival: neither one reactive | -0.175 | -0.236 | -0.113 | <0.001 | -0.172 | -0.234 | -0.11 | <0.001 |
| Pupil at arrival: one reactive | -0.144 | -0.231 | -0.058 | 0.001 | -0.144 | -0.231 | -0.057 | 0.001 |
| SBP at arrival, mmHg | 0 | -0.001 | 0.001 | 0.908 | 0 | -0.001 | 0.001 | 0.864 |
| DBP at arrival, mmHg | -0.001 | -0.002 | 0.001 | 0.247 | -0.001 | -0.002 | 0.001 | 0.248 |
| HR at arrival, beats/min | 0 | -0.001 | 0.001 | 0.981 | 0 | -0.001 | 0.001 | 0.998 |
| RR at arrival, breaths/min | -0.002 | -0.007 | 0.003 | 0.408 | -0.002 | -0.007 | 0.004 | 0.501 |
| SpO2 at arrival, % | 0.006 | 0 | 0.012 | 0.056 | 0.006 | 0 | 0.012 | 0.054 |
| BT at arrival, °C | 0.02 | 0.005 | 0.036 | 0.009 | 0.02 | 0.005 | 0.035 | 0.009 |
| Premorbid mRS, 0 | Ref |  |  |  | Ref |  |  |  |
| Premorbid mRS, 1 | -0.083 | -0.138 | -0.029 | 0.003 | -0.079 | -0.134 | -0.024 | 0.005 |
| Premorbid mRS, 2 or more | -0.173 | -0.232 | -0.114 | <0.001 | -0.172 | -0.232 | -0.113 | <0.001 |
| PMHx: Hypertension | -0.008 | -0.05 | 0.033 | 0.697 | -0.01 | -0.051 | 0.032 | 0.652 |
| PMHx: Diabetes | -0.009 | -0.054 | 0.036 | 0.695 | -0.009 | -0.054 | 0.036 | 0.697 |
| PMHx: Stroke | -0.014 | -0.069 | 0.04 | 0.6 | -0.014 | -0.069 | 0.04 | 0.61 |
| PMHx: Malignancy | -0.079 | -0.137 | -0.021 | 0.008 | -0.08 | -0.138 | -0.022 | 0.007 |
| PMHx: Chronic kidney disease | -0.132 | -0.202 | -0.062 | <0.001 | -0.132 | -0.202 | -0.062 | <0.001 |
| PMHx: Antiplatelet use | 0.013 | -0.036 | 0.062 | 0.591 | 0.013 | -0.037 | 0.062 | 0.616 |
| PMHx: Anticoagulant use | -0.043 | -0.101 | 0.015 | 0.143 | -0.043 | -0.101 | 0.016 | 0.152 |
| PMHx: Non-smoker | Ref |  |  |  | Ref |  |  |  |
| PMHx: Current smoker | -0.015 | -0.069 | 0.039 | 0.584 | -0.016 | -0.069 | 0.038 | 0.568 |
| PMHx: Ex-smoker | 0.035 | -0.033 | 0.103 | 0.317 | 0.036 | -0.033 | 0.104 | 0.307 |
| PMHx: no alcohol use | Ref |  |  |  | Ref |  |  |  |
| PMHx: moderate-to-heavy alcohol use | 0.077 | 0.017 | 0.137 | 0.012 | 0.079 | 0.019 | 0.139 | 0.01 |
| PMHx: infrequent-to-light alcohol use | 0.078 | 0.026 | 0.129 | 0.003 | 0.076 | 0.024 | 0.127 | 0.004 |
| Standard deviation of intercept (group) |  |  |  |  | 0.018 |  |  |  |
| Standard deviation of residual (observation) |  |  |  |  | 0.293 |  |  |  |

**Supplemental Table 5. Full Results of Fixed and Linear Mixed-Effects Models for utility-weighted modified Rankin Scale at 12 months**

|  | **Fixed effect model** | | | | **Mixed effect model** | | | |
| --- | --- | --- | --- | --- | --- | --- | --- | --- |
| **Term** | **Beta** | **CI lower** | **CI upper** | **P-value** | **Beta** | **CI lower** | **CI upper** | **P-value** |
| (Intercept) | -0.24 | -0.908 | 0.428 | 0.481 | -0.235 | -0.903 | 0.432 | 0.489 |
| Age | -0.006 | -0.007 | -0.005 | <0.001 | -0.006 | -0.007 | -0.005 | <0.001 |
| Type of ABI: ICH | Ref |  |  |  | Ref |  |  |  |
| Type of ABI: SAH | 0.104 | 0.05 | 0.158 | <0.001 | 0.11 | 0.056 | 0.164 | <0.001 |
| Type of ABI: TBI | 0.052 | 0.009 | 0.095 | 0.018 | 0.058 | 0.014 | 0.101 | 0.009 |
| Mode of admission: Direct |  |  |  |  |  |  |  |  |
| Mode of admission: In-hospital | -0.041 | -0.153 | 0.072 | 0.48 | -0.052 | -0.165 | 0.061 | 0.37 |
| Mode of admission: Other hospital | 0.053 | 0.01 | 0.097 | 0.017 | 0.052 | 0.008 | 0.096 | 0.021 |
| Onset to arrival time, h | 0 | 0 | 0 | 0.779 | 0 | 0 | 0 | 0.66 |
| GCS score at arrival | 0.036 | 0.03 | 0.041 | <0.001 | 0.036 | 0.03 | 0.042 | <0.001 |
| Pupil at arrival: both reactive | Ref |  |  |  | Ref |  |  |  |
| Pupil at arrival: neither one reactive | -0.151 | -0.212 | -0.09 | <0.001 | -0.146 | -0.207 | -0.085 | <0.001 |
| Pupil at arrival: one reactive | -0.153 | -0.243 | -0.063 | 0.001 | -0.153 | -0.243 | -0.063 | 0.001 |
| SBP at arrival, mmHg | 0 | 0 | 0 | 0.963 | 0 | 0 | 0 | 0.959 |
| DBP at arrival, mmHg | -0.001 | -0.002 | 0 | 0.297 | -0.001 | -0.002 | 0 | 0.315 |
| HR at arrival, beats/min | -0.001 | -0.002 | 0 | 0.199 | -0.001 | -0.002 | 0 | 0.178 |
| RR at arrival, breaths/min | -0.001 | -0.006 | 0.005 | 0.793 | 0 | -0.006 | 0.005 | 0.883 |
| SpO2 at arrival, % | 0.002 | -0.001 | 0.005 | 0.168 | 0.002 | -0.001 | 0.005 | 0.143 |
| BT at arrival, °C | 0.018 | 0.002 | 0.034 | 0.025 | 0.017 | 0.002 | 0.033 | 0.031 |
| Premorbid mRS, 0 | Ref |  |  |  | Ref |  |  |  |
| Premorbid mRS, 1 | -0.106 | -0.161 | -0.05 | <0.001 | -0.095 | -0.152 | -0.039 | 0.001 |
| Premorbid mRS, 2 or more | -0.19 | -0.25 | -0.13 | <0.001 | -0.187 | -0.247 | -0.127 | <0.001 |
| PMHx: Hypertension | -0.008 | -0.051 | 0.034 | 0.704 | -0.011 | -0.053 | 0.032 | 0.625 |
| PMHx: Diabetes | -0.024 | -0.07 | 0.021 | 0.289 | -0.026 | -0.071 | 0.019 | 0.264 |
| PMHx: Stroke | -0.034 | -0.089 | 0.021 | 0.232 | -0.033 | -0.088 | 0.022 | 0.245 |
| PMHx: Malignancy | -0.069 | -0.128 | -0.011 | 0.02 | -0.066 | -0.125 | -0.008 | 0.026 |
| PMHx: Chronic kidney disease | -0.116 | -0.185 | -0.047 | 0.001 | -0.112 | -0.181 | -0.042 | 0.002 |
| PMHx: Antiplatelet use | 0.033 | -0.017 | 0.082 | 0.193 | 0.03 | -0.019 | 0.079 | 0.236 |
| PMHx: Anticoagulant use | -0.005 | -0.065 | 0.055 | 0.87 | -0.003 | -0.063 | 0.057 | 0.915 |
| PMHx: Non-smoker | Ref |  |  |  | Ref |  |  |  |
| PMHx: Current smoker | -0.008 | -0.062 | 0.046 | 0.766 | -0.009 | -0.063 | 0.045 | 0.746 |
| PMHx: Ex-smoker | 0.008 | -0.059 | 0.076 | 0.812 | 0.015 | -0.053 | 0.082 | 0.667 |
| PMHx: no alcohol use | Ref |  |  |  | Ref |  |  |  |
| PMHx: moderate-to-heavy alcohol use | 0.075 | 0.014 | 0.136 | 0.016 | 0.076 | 0.014 | 0.137 | 0.016 |
| PMHx: infrequent-to-light alcohol use | 0.068 | 0.017 | 0.119 | 0.01 | 0.065 | 0.014 | 0.117 | 0.012 |
| Standard deviation of intercept (group) |  |  |  |  | 0.038 |  |  |  |
| Standard deviation of residual (observation) |  |  |  |  | 0.312 |  |  |  |

**Supplemental Table 6. Full Results of Fixed and Mixed-Effects Logistic Regression Models for in-hospital mortality**

|  | **Fixed effect model** | | | | **Mixed effect model** | | | |
| --- | --- | --- | --- | --- | --- | --- | --- | --- |
| **Term** | **aOR** | **CI lower** | **CI upper** | **P-value** | **aOR** | **CI lower** | **CI upper** | **P-value** |
| (Intercept) | -0.675 | -2.372 | 1.01 | 0.433 | -0.675 | -2.362 | 1.012 | 0.433 |
| Mode of admission: Direct to ER | Ref |  |  |  | Ref |  |  |  |
| Mode of admission: In-hospital (except ER) | 0.68 | -0.341 | 1.701 | 0.19 | 0.68 | -0.338 | 1.698 | 0.19 |
| Mode of admission: From other hospital to ER | -0.47 | -1.086 | 0.128 | 0.128 | -0.47 | -1.076 | 0.135 | 0.128 |
| Onset to arrival time, ≥ 6h | Ref |  |  |  | Ref |  |  |  |
| Onset to arrival time, 0 - 1h | 0.342 | -0.354 | 1.061 | 0.342 | 0.342 | -0.364 | 1.047 | 0.342 |
| Onset to arrival time, 1 - 3h | 0.472 | -0.189 | 1.157 | 0.168 | 0.472 | -0.198 | 1.143 | 0.168 |
| Onset to arrival time, 3 - 6h | 0.024 | -0.812 | 0.84 | 0.955 | 0.024 | -0.798 | 0.846 | 0.955 |
| arrival_gcs_score_num | -0.216 | -0.277 | -0.156 | <0.001 | -0.216 | -0.276 | -0.156 | <0.001 |
| Pupil at arrival: both reactive | Ref |  |  |  | Ref |  |  |  |
| Pupil at arrival: neither one reactive | 1.44 | 0.922 | 1.97 | <0.001 | 1.44 | 0.917 | 1.964 | <0.001 |
| Pupil at arrival: one reactive | 0.513 | -0.347 | 1.304 | 0.221 | 0.513 | -0.308 | 1.333 | 0.221 |
| SBP at arrival, mmHg | -0.003 | -0.009 | 0.002 | 0.251 | -0.003 | -0.009 | 0.002 | 0.251 |
| HR at arrival, beats/min | -0.002 | -0.011 | 0.008 | 0.716 | -0.002 | -0.011 | 0.008 | 0.716 |
| RR at arrival, breaths/min | 0.038 | -0.01 | 0.085 | 0.117 | 0.038 | -0.009 | 0.085 | 0.117 |
| Premorbid mRS, 0 | Ref |  |  |  | Ref |  |  |  |
| Premorbid mRS, 1 | 0.456 | -0.093 | 0.997 | 0.1 | 0.456 | -0.088 | 1 | 0.1 |
| Premorbid mRS, 2 or more | 0.064 | -0.527 | 0.638 | 0.828 | 0.064 | -0.517 | 0.645 | 0.828 |
| PMHx: Malignancy | 0.969 | 0.401 | 1.533 | 0.001 | 0.969 | 0.404 | 1.533 | 0.001 |
| PMHx: Chronic kidney disease | 0.545 | -0.188 | 1.252 | 0.136 | 0.545 | -0.172 | 1.263 | 0.136 |
| PMHx: Anticoagulant use | 0.13 | -0.464 | 0.708 | 0.663 | 0.13 | -0.455 | 0.715 | 0.663 |
| PMHx: Non-smoker | Ref |  |  |  | Ref |  |  |  |
| PMHx: Current smoker | -0.047 | -0.68 | 0.563 | 0.881 | -0.047 | -0.668 | 0.573 | 0.881 |
| PMHx: Ex-smoker | 0.213 | -0.53 | 0.922 | 0.565 | 0.213 | -0.511 | 0.937 | 0.565 |
| PMHx: no alcohol use | Ref |  |  |  | Ref |  |  |  |
| PMHx: moderate-to-heavy alcohol use | -0.597 | -1.313 | 0.086 | 0.094 | -0.597 | -1.294 | 0.101 | 0.094 |
| PMHx: infrequent-to-light alcohol use | -0.503 | -1.098 | 0.062 | 0.088 | -0.503 | -1.082 | 0.075 | 0.088 |
| Standard deviation of intercept (group) |  |  |  |  | 0 |  |  |  |

aOR: adjusted odds ratio, Ref: Reference category, GCS: Glasgow Coma Scale, SBP: Systolic Blood Pressure, HR: Heart Rate, RR: Respiratory Rate, mRS: Modified Rankin Scale, ER: emergency room and CI lower and CI upper refer to the lower and upper bounds of the 95% confidence interval, respectively.

**Supplemental Table 7. Full Results of Fixed and Linear Mixed-Effects Models for length of neurocritical care unit stay**

|  | **Fixed effect model** | | | | **Mixed effect model** | | | |
| --- | --- | --- | --- | --- | --- | --- | --- | --- |
| **Term** | **Beta** | **CI lower** | **CI upper** | **P-value** | **Beta** | **CI lower** | **CI upper** | **P-value** |
| (Intercept) | 6.43 | 3.111 | 9.748 | <0.001 | 6.28 | 2.961 | 9.6 | <0.001 |
| Type of ABI: ICH | Ref |  |  |  | Ref |  |  |  |
| Type of ABI: SAH | 0.35 | 0.145 | 0.556 | 0.001 | 0.366 | 0.161 | 0.572 | <0.001 |
| Type of ABI: TBI | -0.176 | -0.338 | -0.015 | 0.033 | -0.156 | -0.32 | 0.007 | 0.06 |
| Mode of admission: Direct to ER | Ref |  |  |  | Ref |  |  |  |
| Mode of admission: In-hospital (except ER) | 0.148 | -0.303 | 0.599 | 0.52 | 0.112 | -0.339 | 0.563 | 0.625 |
| Mode of admission: From other hospital to ER | 0.199 | 0.007 | 0.392 | 0.043 | 0.209 | 0.015 | 0.404 | 0.035 |
| Onset to arrival time, ≥ 6h | Ref |  |  |  | Ref |  |  |  |
| Onset to arrival time, 0 - 1h | 0.236 | 0.012 | 0.46 | 0.039 | 0.247 | 0.023 | 0.47 | 0.031 |
| Onset to arrival time, 1 - 3h | 0.294 | 0.084 | 0.505 | 0.006 | 0.323 | 0.11 | 0.536 | 0.003 |
| Onset to arrival time, 3 - 6h | 0.24 | -0.001 | 0.48 | 0.052 | 0.236 | -0.005 | 0.477 | 0.055 |
| GCS score at arrival | -0.109 | -0.132 | -0.086 | <0.001 | -0.108 | -0.132 | -0.084 | <0.001 |
| Pupil at arrival: both reactive | Ref |  |  |  | Ref |  |  |  |
| Pupil at arrival: neither one reactive | -0.048 | -0.286 | 0.189 | 0.691 | -0.04 | -0.277 | 0.198 | 0.744 |
| Pupil at arrival: one reactive | 0.322 | -0.015 | 0.659 | 0.061 | 0.278 | -0.06 | 0.615 | 0.107 |
| HR at arrival, beats/min | -0.001 | -0.005 | 0.002 | 0.492 | -0.002 | -0.005 | 0.002 | 0.412 |
| RR at arrival, breaths/min | 0.007 | -0.013 | 0.028 | 0.496 | 0.009 | -0.013 | 0.03 | 0.416 |
| SpO2 at arrival, % | -0.015 | -0.038 | 0.008 | 0.202 | -0.016 | -0.039 | 0.008 | 0.188 |
| BT at arrival, °C | -0.047 | -0.108 | 0.014 | 0.134 | -0.042 | -0.103 | 0.019 | 0.176 |
| PMHx: Diabetes | 0.169 | 0 | 0.338 | 0.05 | 0.168 | 0 | 0.337 | 0.05 |
| PMHx: Malignancy | 0.072 | -0.149 | 0.292 | 0.524 | 0.052 | -0.169 | 0.273 | 0.644 |
| PMHx: Chronic kidney disease | 0.401 | 0.128 | 0.673 | 0.004 | 0.377 | 0.105 | 0.65 | 0.007 |
| PMHx: Antiplatelet use | -0.018 | -0.195 | 0.16 | 0.846 | -0.029 | -0.206 | 0.148 | 0.747 |
| Standard deviation of intercept (group) |  |  |  |  | 0.147 |  |  |  |
| Standard deviation of residual (observation) |  |  |  |  | 1.176 |  |  |  |

Ref: Reference category, ABI: Acute Brain Injury, ER: emergency room, GCS: Glasgow Coma Scale, HR: Heart Rate, RR: Respiratory Rate, HR: Heart Rate, SpO2: Oxygen Saturation, BT: Body Temperature, PMHx: Past Medical History.

CI lower and CI upper refer to the lower and upper bounds of the 95% confidence interval, respectively.

**Supplemental Table 8. Full Results of Fixed and Mixed-Effects Logistic Regression Models for tracheostomy**

|  | **Fixed effect model** | | | | **Mixed effect model** | | | |
| --- | --- | --- | --- | --- | --- | --- | --- | --- |
| **Term** | **aOR** | **CI lower** | **CI upper** | **P-value** | **aOR** | **CI lower** | **CI upper** | **P-value** |
| (Intercept) | 5.906 | -2.226 | 13.331 | 0.128 | 6.112 | -1.525 | 13.749 | 0.117 |
| Age, years | -0.015 | -0.03 | -0.001 | 0.03 | -0.015 | -0.03 | -0.001 | 0.033 |
| Mode of admission: Direct to ER | Ref |  |  |  | Ref |  |  |  |
| Mode of admission: In-hospital (except ER) | 1.341 | 0.402 | 2.272 | 0.005 | 1.254 | 0.308 | 2.2 | 0.009 |
| Mode of admission: From other hospital to ER | 0.306 | -0.273 | 0.879 | 0.296 | 0.46 | -0.151 | 1.071 | 0.14 |
| Onset to arrival time, ≥ 6h | Ref |  |  |  | Ref |  |  |  |
| Onset to arrival time, 0 - 1h | 0.763 | 0.046 | 1.517 | 0.041 | 0.871 | 0.12 | 1.622 | 0.023 |
| Onset to arrival time, 1 - 3h | 0.963 | 0.302 | 1.662 | 0.005 | 1.125 | 0.416 | 1.834 | 0.002 |
| Onset to arrival time, 3 - 6h | 0.585 | -0.225 | 1.392 | 0.153 | 0.642 | -0.165 | 1.45 | 0.119 |
| GCS score at arrival | -0.196 | -0.257 | -0.137 | <0.001 | -0.19 | -0.252 | -0.127 | <0.001 |
| Pupil at arrival: both reactive | Ref |  |  |  | Ref |  |  |  |
| Pupil at arrival: neither one reactive | -0.087 | -0.636 | 0.459 | 0.754 | -0.034 | -0.589 | 0.521 | 0.904 |
| Pupil at arrival: one reactive | 0.361 | -0.449 | 1.109 | 0.361 | 0.373 | -0.408 | 1.155 | 0.349 |
| SBP at arrival, mmHg | 0.001 | -0.005 | 0.007 | 0.717 | 0.001 | -0.005 | 0.007 | 0.74 |
| HR at arrival, beats/min | -0.003 | -0.013 | 0.006 | 0.507 | -0.004 | -0.013 | 0.006 | 0.487 |
| RR at arrival, breaths/min | 0.011 | -0.037 | 0.058 | 0.665 | 0.012 | -0.038 | 0.062 | 0.641 |
| SpO2 at arrival, % | -0.041 | -0.093 | 0.009 | 0.112 | -0.047 | -0.099 | 0.005 | 0.075 |
| BT at arrival, °C | -0.07 | -0.201 | 0.103 | 0.345 | -0.065 | -0.208 | 0.077 | 0.368 |
| Premorbid mRS, 0 | Ref |  |  |  | Ref |  |  |  |
| Premorbid mRS, 1 | -0.447 | -1.101 | 0.159 | 0.162 | -0.343 | -0.991 | 0.305 | 0.299 |
| Premorbid mRS, 2 or more | 0.797 | 0.209 | 1.379 | 0.007 | 0.781 | 0.188 | 1.374 | 0.01 |
| PMHx: Malignancy | -0.011 | -0.657 | 0.593 | 0.973 | -0.07 | -0.7 | 0.561 | 0.828 |
| PMHx: Hypertension | 0.497 | 0.033 | 0.972 | 0.038 | 0.497 | 0.025 | 0.968 | 0.039 |
| PMHx: Diabetes | 0.343 | -0.129 | 0.807 | 0.15 | 0.361 | -0.109 | 0.831 | 0.132 |
| PMHx: Stroke | 0.335 | -0.212 | 0.866 | 0.222 | 0.277 | -0.269 | 0.824 | 0.32 |
| PMHx: Chronic kidney disease | 0.738 | 0.064 | 1.382 | 0.028 | 0.688 | 0.025 | 1.351 | 0.042 |
| Standard deviation of intercept (group) |  |  |  |  | 0.319 |  |  |  |

aOR: adjusted odds ratio, Ref: Reference category, ER: emergency room, GCS: Glasgow Coma Scale, SBP: Systolic Blood Pressure, HR: Heart Rate, RR: Respiratory Rate, SpO2: Oxygen saturation, BT: body temperature, mRS: modified Rankin Scale, PMHx: past medical history.

CI lower and CI upper refer to the lower and upper bounds of the 95% confidence interval, respectively.
